# The Chemical Relationship Among Beta-Lactam Antibiotics and Potential Impacts on Reactivity and Decomposition

**DOI:** 10.3389/fmicb.2022.807955

**Published:** 2022-03-24

**Authors:** Jonathan Turner, Alyssa Muraoka, Michael Bedenbaugh, Blaine Childress, Lauren Pernot, Mark Wiencek, Yuri K. Peterson

**Affiliations:** ^1^College of Medicine, Medical University of South Carolina, Charleston, SC, United States; ^2^College of Pharmacy, Medical University of South Carolina, Charleston, SC, United States; ^3^Intramed Plus, West Columbia, SC, United States; ^4^South Carolina Research Authority, Greenville, SC, United States; ^5^Contec, Inc., Spartanburg, SC, United States

**Keywords:** hydrolysis, decomposition, chemical informatics, lactam antibiotics, antibiotic allergy (including penicillin and cephalosporin β-lactams), antimicrobial activity, deactivation

## Abstract

Beta-lactam antibiotics remain one of the most commonly prescribed drug classes, but they are limited by their propensity to cause hypersensitivity reactions (e.g., from allergy to anaphylaxis) as well as by the emergence of bacteria with a myriad of resistance mechanisms such as β-lactamases. While development efforts continue to focus on overcoming resistance, there are ongoing concerns regarding cross-contamination of β-lactams during manufacturing and compounding of these drugs. Additionally, there is a need to reduce levels of drugs such as β-lactam antibiotics in waste-water to mitigate the risk of environmental exposure. To help address future development of effective remediation chemistries and processes, it is desired to better understand the structural relationship among the most common β-lactams. This study includes the creation of a class-wide structural ordering of the entire β-lactam series, including both United States Food and Drug Association (US-FDA)-approved drugs and experimental therapies. The result is a structural relational map: the “Lactamome,” which positions each substance according to architecture and chemical end-group. We utilized a novel method to compare the structural relationships of β-lactam antibiotics among the radial cladogram and describe the positioning with respect to efficacy, resistance to hydrolysis, reported hypersensitivity, and Woodward height. The resulting classification scheme may help with the development of broad-spectrum treatments that reduce the risk of occupational exposure and negative environmental impacts, assist practitioners with avoiding adverse patient reactions, and help direct future drug research.

## Historical Overview

**FIGURE 1:**
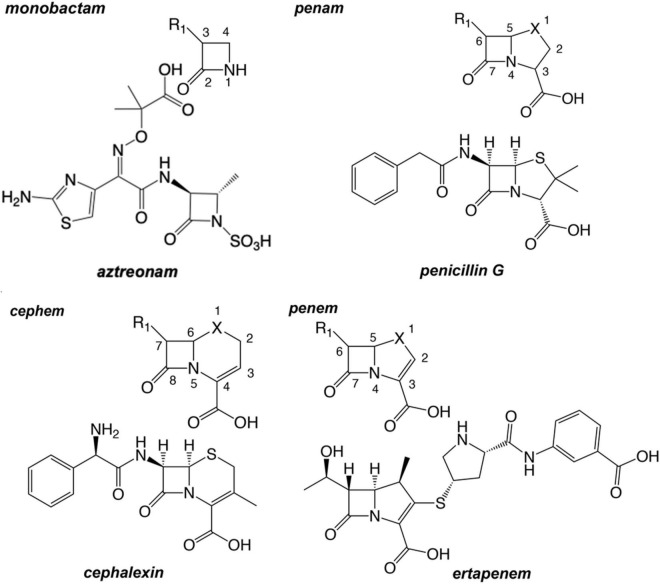
The chemical graphs and scaffold of primary examples of major β-lactam antibiotic classes. Common atom numbering scheme of the chemotype is depicted and oriented above the example β-lactam ring. The X may be a carbon, an oxygen atom, or a sulfur atom. For example, penem structures with X=C are “carbapenems”; penem structures with X=O are “oxapenems”; and penem structures with X=S are “thiapenems,” or more commonly, simply “penems.”

Since the first clinical uses of penicillin G in the 1930s and 1940s, β-lactam antimicrobials have enjoyed marked success in the treatment of bacterial infections. By 1944 penicillin was being manufactured at the rate of over a billion doses a year (Aldridge et al., 1999). Shortly after the introduction of the penam penicillin, the first chemical compounds of the cephem group were isolated from the fungus *Cephalosporium acremonium* by Giuseppe Brotzu in 1948, when crude filtrates of a culture were found to inhibit the growth of *Staphylococcus aureus* (Singh and Arrieta, 1999; Foye et al., 2008). The first cephem, cephalothin, became available for patients in the United States as a parenteral drug in 1964 (Greenwood, 2008). Interest in β-lactam antibiotics spurred scientists to explore a breadth of structural derivatives and platforms well beyond the above-mentioned natural substances in efforts to expand the spectrum. Those synthetic efforts have resulted in four major β-lactam classes: monobactam, penams, penems, and cephems (Figure 1).

## Structural Platforms and β-Lactam Classes

**FIGURE 2:**
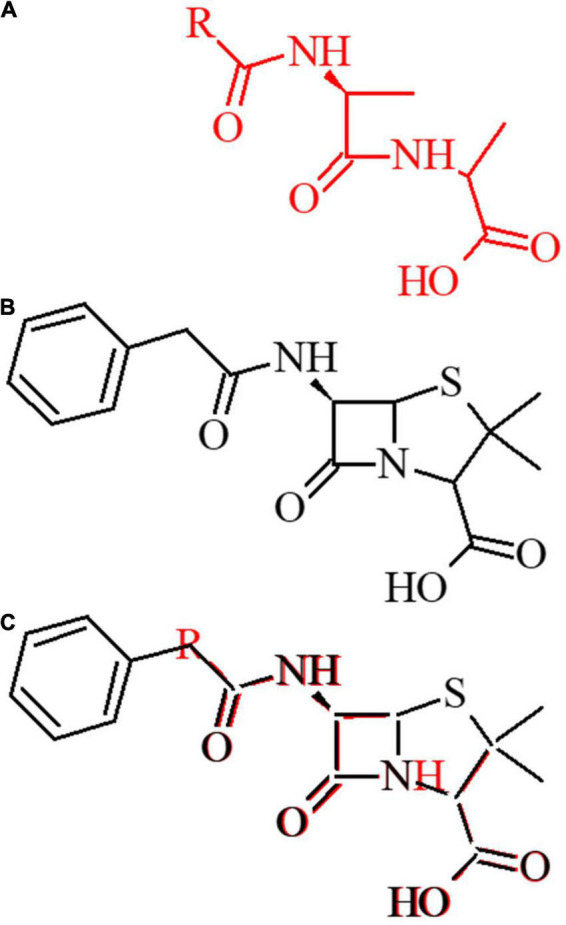
Structural basis of lactam activity. **(A)** The D-Ala-D-Ala amino acid is depicted in red. **(B)** The 1st generation penam ampicillin is depicted in black. **(C)** D-Ala-D-Ala dipeptide overlay with the β-lactam ampicillin antibiotic.

The historical development process as well as structural and functional (e.g., spectra of activity) differences among the various classes of β-lactams are described in detail in several excellent reviews (Bush and Bradford, 2016; Ochoa-Aguilar et al., 2016; Lima et al., 2020). Aside from the impacts resulting from electron withdrawing substituents by different R groups, the key structural features of all β-lactam antibiotics include (1) the 2-azetidinone, a 4-membered cyclic amide required for their characteristic target acylation, (2) the level of cyclic amide ring strain imposed by bicyclic structural designs, and (3) an acidic moiety critical to their recognition by the transpeptidase active site (Figure 2).

While many previous studies have investigated the intricacies of drug binding and the impacts on efficacy and resistance, the relationship of molecular structure on the ability of chemical compounds and processes to decontaminate β-lactam residues from manufacturing and compounding facilities as well as terrestrial and aquatic environments has received less attention. Because of their perceived or actual potential to cause allergic responses in a substantial portion of the host population, governmental guidelines and regulations involve special precautions to reduce the risk of cross-contamination from β-lactams (Food and Drug Administration [FDA], 2013). The chemicals that might be candidates for decontamination of surfaces in facilities or for waste-water treatment should be effective against a broad range of drugs at levels that are already employed for disinfection and be readily biodegradable themselves.

As such, it was desired to assess the structural relationships among β-lactams to understand if a subset of drugs can serve as a proxy for others during development and testing. As a first step toward this attempt of a “read-across,” the most widely used β-lactam antibiotics have been reviewed, molecularly modeled, and structurally organized into relational maps termed a “Lactamome.”

### β-Lactam Classes

The **monocyclic β-lactams** (or monobactam) class represents the simplest structural platform among these antibiotics. The monocyclic platform of this b-lactam was first isolated from *Chromobacterium violaceum* (Asai et al., 1981; Imada et al., 1981; Sykes et al., 1981; Decuyper et al., 2018; Goldberg et al., 2021). There are three major monocyclic β-lactams: aztreonam, tigemonam, nocardicin A, and two minor monocyclic β-lactams: carumonam (only approved in Japan) and tabtoxin. Tabtoxin, the only member retaining a nitrogen-hydrogen bond, is uniquely a glutamine synthetase inhibitor with lower activity against penicillin-binding proteins (PBPs). The others possess an *N*-sulfonic acid moiety or *N*-acetic acid moiety. Aztreonam (Figure 1), currently the only monocyclic β-lactams clinically used in the United States, exhibits potent inhibition of Gram-negative PBPs (Georgopapadakou et al., 1982; Sykes et al., 1982; Rittenbury, 1990; Cunha, 1993) but finds limited clinical utility owing to its lack of activity against Gram-positive or anaerobic bacteria. Because of its unique monocyclic β-lactam structure, aztreonam is thought to have no cross-sensitivity to patients allergic to penicillin (Gaeta et al., 2015). Currently there is pre-clinical research in aztreonam combination with other β-lactams and diazabicyclooctane monocyclic β-lactams against MDR/XDR Gram-negative bacteria (Bassetti et al., 2021; Bhatnagar et al., 2021). A second generation of monocyclic β-lactams is also under late stage clinical development (Osborn et al., 2019).

**Penams** are a class of β-lactams possessing an azabicyclo[3.2.0]heptane ring system, a carboxylic acid moiety at C3, and a sulfur atom at position one (Figure 1). The prototypic and most well-known penam is penicillin G (PNG or benzylpenicillin), isolated from *Penicillium chrysogenum* (Fleming, 2001). The other commonly used natural product penam is penicillin V, or phenoxymethylpenicillin. In the absence of β-lactamases, the natural penicillins show good Gram-positive activity but fail to inhibit the growth of many Gram-negative organisms (Libby and Holmberg, 1945; Eagle, 1946; Eagle and Technical Assistance of Arlyne D. Musselman, 1947; Nell and Hill, 1947; Thomsen and Larsen, 1962). A great deal of creative synthetic chemistry has been directed to the penam platform to modulate the spectrum of this class, predominantly *via* modifications at the C6 acylamino group (R_1_ side chain). Clinically useful penam derivatives have geminal dimethyl groups at C2, except tazobactam which is used in combination therapies as a β-lactamase inhibitor. The penam amoxicillin is one of the most often prescribed antibiotics in the United States, many other countries, and in veterinary medicine (Tao et al., 2019).

**Penems** are direct analogs of penams but with 2,3-unsaturation within the fused five-membered ring (Figure 1). The thiapenems, which contain a sulfur atom in the fused ring at position one, do not exist naturally but were synthesized in 1978 by chemists including R.B. Woodward (Ernest et al., 1978). The penem class is one of a few β-lactam antibiotics still being researched, and includes faropenem, ritipenem, and sulopenem (Batchelder et al., 2020; Butler et al., 2022; Sato et al., 2022). While no members of the thiapenem class are currently used in United States clinical settings, safety and efficacy data required for FDA approval are forthcoming. However, there are several FDA-approved members of a subclass of penems called carbapenems, which possess a carbon atom in place of the sulfur “X” atom. The structure of ertapenem is shown in Figure 1 as an example of this class. The C6 hydroxyethyl substituent possessed by members of the penem class represents a significant departure from penams and cephems, which generally exhibit large and variable aminoacyl substituents at C6. Differences in activity and spectrum among compounds of this class are influenced by *cis* alignment of the vicinal R1 hydroxyl and C6 hydrogen and dictated by the C2 substituent. Given their broad spectrum, as well as the β-lactamase stability afforded by their C6 *R*-hydroxyethyl side chain and *trans* C5-C6 hydrogen geometry (Basker et al., 1980), the carbapenems have found most of their clinical utility as antimicrobials for infections with multidrug-resistant organisms.

**Cephems**, also known as cephalosporins, differ from penems by having a dihydrothiazine ring fused to the β-lactam ring (Hughes, 2017a,b). First generation cephems generally possess an aromatic acylamino side chain at C7 and exhibit potent Gram-positive activity (Godzeski et al., 1963; Spencer et al., 1966a,b; Ryan et al., 1969; Hsieh and Ho, 1975).

As cephems entered a second generation, drug developers explored other substituents at C7, such as the replacement of the acetoxy group with various nucleophiles (i.e., carbamates, heterocyclic mercaptans, and pyridines). These cephems exhibit broader spectrum than their earlier counterparts, with some showing potent inhibition of Gram-negatives (Eykyn et al., 1973; Kosmidis et al., 1973; Hamilton-Miller et al., 1974; Neu, 1974a,b; Nomura et al., 1974; Wallick and Hendlin, 1974; Tsuchiya et al., 1978). The broad cephem class also contains the cephamycin (and cephalosporin) class which also has the cephem nucleus and includes the second generation cefoxitin and cefotetan (Kosmidis et al., 1973; Neu, 1974b; Wallick and Hendlin, 1974).

In third generation designs, the aryl group was modified to an aminothiazole, a change found to enhance Gram-negative activity. One notable outlier is cefoperazone, which borrows its C7 acylamino group from the ureidopenicillin piperacillin. This group exhibits not only further expanded spectrum compared to previous generations, but maintains stability to serine β-lactamases (Sosna et al., 1978; Kojo et al., 1979; Matsubara et al., 1979; O’Callaghan et al., 1980; Reiner et al., 1980; Shannon et al., 1980; Verbist and Verhaegen, 1980; Wise et al., 1980; Neu et al., 1981, 1984, 1989; Kamimura et al., 1984; Inamoto et al., 1988, 1990a,b; Yokoo et al., 1991).

With fourth generation cephems, quaternary nitrogen substituents were incorporated at C3 to enhance passive transport through the Gram-negative outer membrane, decrease affinity for β-lactamases, and promote departure of the leaving group (Machka and Braveny, 1983; Seibert et al., 1983; Khan et al., 1984; Kessler et al., 1985; Limbert et al., 1991; Chin et al., 1992; Hancock and Bellido, 1992).

The fifth generation of cephems was specifically engineered to combat various resistance problems while attempting to maintain a broad spectrum of activity (Entenza et al., 2002; Wootton et al., 2002; Zbinden et al., 2002; Ishikawa et al., 2003; Iizawa et al., 2004; Issa et al., 2004; Sader et al., 2005; Takeda et al., 2007a,b; Toda et al., 2008; Ito et al., 2016; Kohira et al., 2016; Dobias et al., 2017; Aoki et al., 2018). In general, these cephems maintain the alkoxyimino group, exchange the aminothiazole for an aminothiadiazole, and install conjugation or extended ring systems in their C3 substituents.

A unique and small modification to the cephem class is the **oxacephem**, which possesses an azabicyclo[4.2.0]oct-2-ene ring system, a carboxylic acid moiety at C4, and, as the name suggests, an oxygen at position one in place of sulfur. The major oxacephems are moxalactam (latamoxef) and flomoxef. These compounds were developed to reduce blood coagulation defects and, more specifically, target the bacterial genus *Nocardia* (Yazawa et al., 1989; Cazzola et al., 1993).

**b-lactamase inhibitors** include subclasses of penam β-lactams resistant to hydrolysis by β-lactamases. While the success of β-lactam antibiotics has been extraordinary, their long-term usage has collided with the evolution of resistant microbial strains. Myriad resistance determinants can contribute to such resistance, but β-lactamases are of particular concern due to their high catalytic efficiency and their potential for rapid dissemination by horizontal transfer on plasmids. Major β-lactam-based β-lactamase inhibitors include sulbactam, clavulanate, and tazobactam. These β-lactam molecules exhibit little direct antibacterial properties by themselves but serve a sacrificial role by tenaciously binding to β-lactamases to thereby enable the unimpeded performance of a partner β-lactam such as amoxicillin (Pechere and Kohler, 1999; Shapiro et al., 2021).

### Mechanism of Activity

β-lactam antibiotics act through structural mimicry of the D-alanine-D-alanine motif in peptidoglycans of the bacterial cell wall (Figure 2). They inhibit bacterial transpeptidases which catalyze the cross-linkage of peptidoglycan through the formation of isopeptide bonds (Goffin and Ghuysen, 1998; Macheboeuf et al., 2006; Sauvage et al., 2008). Peptidoglycan is an essential component of the bacterial cell wall and plays major roles in protecting bacteria from environmental stress, ensuring osmotic stability. Cell wall degradation and resynthesis are critical to bacterial cell growth and division. The major target of β-lactams are PBPs, which are essential peptide cross-linking enzymes and are essential for peptidoglycan synthesis. In the presence of a β-lactam, the serine nucleophile attacks the lactam carbonyl, resulting in a stable acyl-enzyme complex (Yocum et al., 1979; Waxman et al., 1980). This action interrupts the integrity of the bacterial cell wall, diminishing the capacity for growth and division and removing protection from osmotic or tensile stress. A second major mechanism of action, specific to carbapenem β-lactams are the L,D-transpeptidases. For example, faropenem efficacy against mycobacteria is due to L,D transpeptidase covalent adduct formation (Lohans et al., 2019; Lu et al., 2020).

While target affinity and site accumulation are important factors, the efficacy of β-lactams has canonically been dominated by their propensity for chemical acylation, and therefore, a consequence of their ease of entry into the PBP site (i.e., a diffusion-limited kinetic process). This chemical reactivity is strongly related to the geometry at the β-lactam nitrogen and the degree of ring strain. In monocyclic structures, such as monobactams, the amide bond is stabilized by conjugation between the nitrogen lone pair and the carbonyl group. In bicyclic structures, the amide stabilization is attenuated by a third bonding partner, which creates a trigonal pyramidal (sp^3^) nitrogen geometry. The reduced amide resonance conferred by these bicyclic systems affords increased carbonyl electrophilicity and higher intrinsic reactivity to nucleophiles and nucleophile-containing enzymes such as transpeptidases, β-lactamases, and metallo-β-lactamases (Green et al., 1965; Morin et al., 1969; Sweet and Dahl, 1970; Glover and Rosser, 2012; Szostak et al., 2015; Ismalaj, 2022).

**FIGURE 3:**
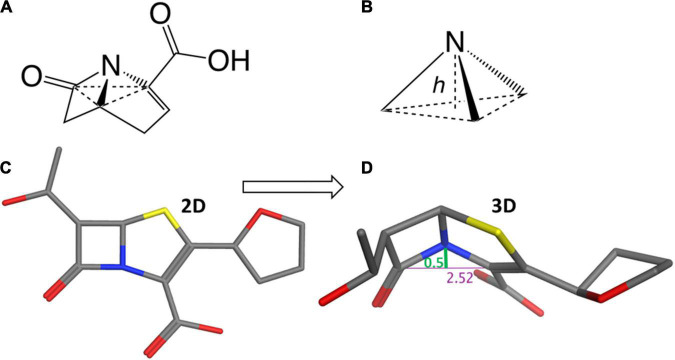
Structural flexibility of β-lactams and the h-Woodward coefficient. **(A)** General bicyclic β-lactam structure rendered to show pyramidal nitrogen. **(B)** Depiction of h-Woodward, defined by height of nitrogen to trigonal pyramid ligand base. **(C)** Faropenem depicted in stick form. **(D)** Faropenem energy minimized (using MOE with the Amber10:ETH forcefield) and angle of the bent nitrogen bonds showing the h-Woodward of 0.5 Å.

Considering the trigonal pyramid, defined by the nitrogen (i.e., the apex) and its three bond-pair substituents (i.e., the base) (Figure 3), the distortion from a planar (sp^2^) amide can be defined by a parameter known as the Woodward height (*h*-Woodward) (Ernest et al., 1978; Lang et al., 1978; Woodward, 1980; Meng et al., 2021; Ismalaj, 2022). Monocyclic monobactams, having an essentially planar geometry at nitrogen, tend to be the least reactive of the β-lactams with negligible *h*-Woodward values (Diaz et al., 2002).

Cephems are sterically predisposed toward Woodward heights of about 0.2–0.3 Å, due to conformational flexibility afforded by their six-membered ring fusion. However, cephalosporins have unique features that contribute to their chemical reactivities beyond *h*-Woodward. A competitive enamine resonance resulting from the delocalization of the nitrogen’s lone electron pair into the π electron system of the dihydrothiazine ring contributes to attenuated amide resonance (Morin et al., 1969; Sweet and Dahl, 1970). In the setting of this resonance, heteroatomic moieties at the C3 position can act as leaving groups upon ring opening. Whether or not it is a leaving group, the C3 substituent can participate in long range inductive effects with the β-lactam amide moiety–strongly electron-withdrawing groups are postulated to enhance the electrophilicity of the carbonyl (Morin et al., 1969; Boyd et al., 1975; Boyd, 1984).

Penams exhibit still larger *h*-Woodward (∼0.4 Å) values than cephams due to the additional strain imposed by five-membered ring fusion, and as such exhibit higher intrinsic reactivity to nucleophiles. Interestingly, molecules of the penam class can exist in two distinct conformational states, one in which the C3 carboxylate is in an equatorial position in relation to the ring system and one in which it is axial (Abrahamsson et al., 1963; James et al., 1968; Dexter and van der Veen, 1978).

Structural analysis reveals carbapenems and thiapenems as having *h*-Woodward values of 0.50–0.60 Å; these drugs are recognized as among the most reactive β-lactams. Such higher values may be attributed to the additional geometric constraints introduced by the unsaturation between C2 and C3 of the pyrrolidine ring, as well as replacement of the position one sulfur atom with a carbon atom in carbapenems. Their rapid acylation of PBPs have been ascribed to this highly activated β-lactam ring system. Such ring opening propensity has been shown by examining the kinetics of base hydrolysis (Woodward, 1980). Results indicate the penems are the most readily hydrolyzed of known β-lactams (Knox, 1961; Frere et al., 1975; Graves-Woodward and Pratt, 1998; Hujer et al., 2005; Silvaggi et al., 2005; Jeong et al., 2018).

## Results and Discussion

### Chemical Similarity Among β*-*Lactams

**FIGURE 4:**
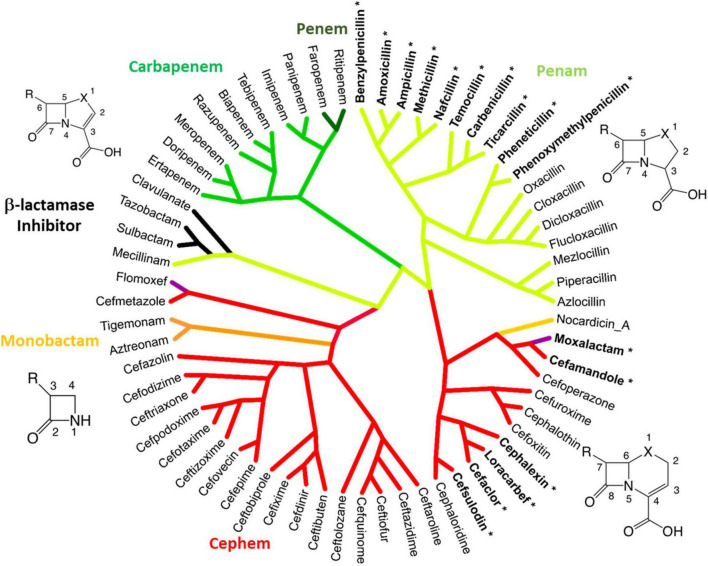
The Lactamome: Radial chemogram of major β-lactams by chemical similarity. Clustering used hierarchical complete linkage MAACS-keyed Tanimoto coefficient. Clustering was performed using ChemMine and visualized using Dendroscope (Huson et al., 2007; Backman et al., 2011). Colors indicate major chemotypes. Note that the oxacephems: moxalactam and flomoxef, are colored purple. Bolded lactams marked with an asterisk indicate drugs with high allergy potential (>10%) relating to the R_1_ benzyl groups.

Considering the evolution and history of β-lactams, this study utilized a novel method to examine the chemical similarity and interrelatedness of β-lactam antibiotics. To perform this analysis, the complete pairwise chemical similarity was calculated among β-lactam drugs approved by the FDA or compounds in late-stage clinical trials, to create a radial cladogram analogous to the common method of how genes, strains or species are compared in a phylogeny. The analysis was performed using the industry standard Tanimoto coefficient similarity score calculated from keyed organic chemical groups using ChemMine and visualized using Dendroscope (Huson et al., 2007; Backman et al., 2011; Peterson et al., 2013). The resulting “Lactamome” (Figure 4) was depicted with benzylpenicillin (penicillin G) as the top-most molecule, owing to its place as a foundational member. The resulting circular chemogram (dendrogram of chemicals) was color-coded according to known β-lactam chemical classes. The utility of chemical clustering is that relationships are unbiased and based solely on organic chemistry. Broadly speaking, the Lactamome shows the major penam and cephem clusters in relation to the minor monobactam, β-lactam-based β-lactamase inhibitor, oxacephem, carbapenem and penem classes. The positioning reflects the similarity of the atomic arrangements in fused or unfused ring systems in relation to the β-lactam foundation, coupled with the electronic effects of substituent end groups. Beginning with benzylpenicillin, penems cluster in four distinct subclasses. This is followed by two major branches and five subgroups of the cephem class.

Monobactams fit in their own small cluster that branches from the cephem class. However, nocardicin has substituents with structural similarity to moxalactam and was positioned apart from other monobactams despite its single ring platform. β-lactamase inhibitors based on the core β-lactam structure (sulbactam, clavulanate, and tazobactam) make up their own cluster. Interestingly along with the penam mecillinam, members of this branch are highly related *via* their core chemistry (3,3-dimethyl-7-oxo-4-thia-1-azabicyclo[3.2.0]heptane-2-carboxylic acid, Figure 1 penam). Oxacephems (moxalactam and flomoxef) are uniquely located as branching off the cephem class.

**FIGURE 5:**
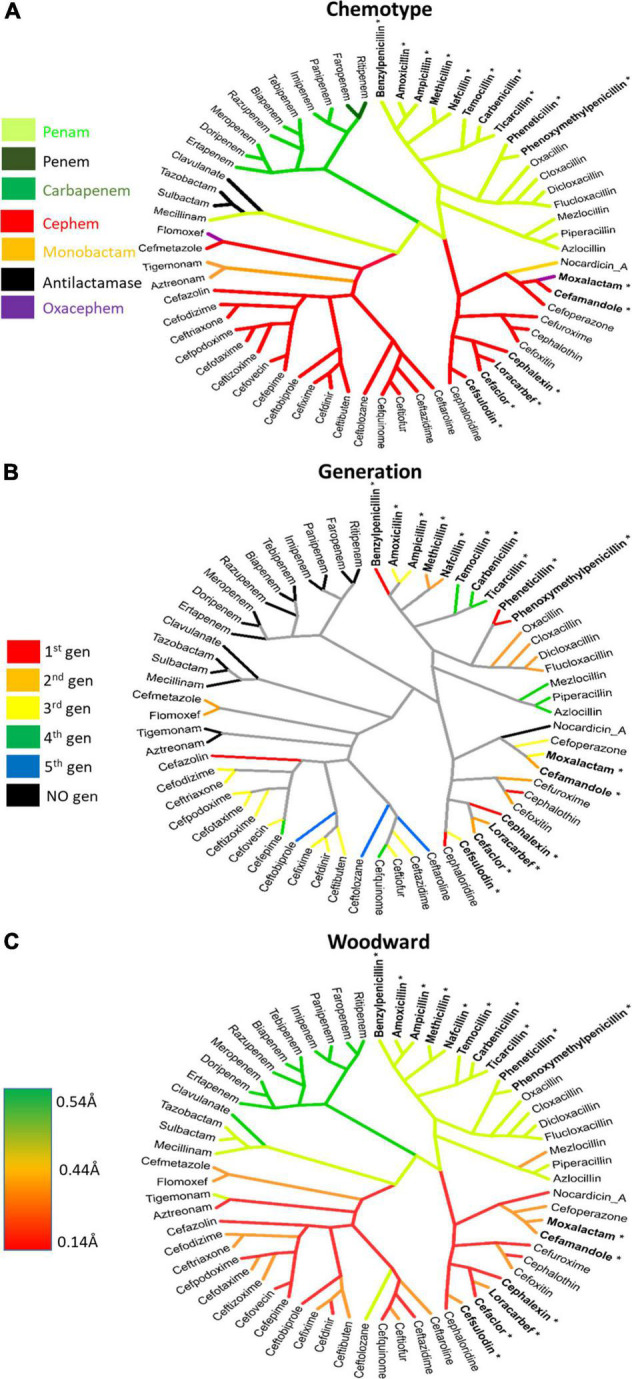
Lactamomes depicting **(A)** chemotype, **(B)** compound generation, and **(C)** *h*-Woodward values. **(A)** The chemotype is a reproduction of Figure 4 to aid comparisons to the other figures. **(B)** The generational lactamome assigns generation levels (1st–5th) to individual drugs based on how they most currently referenced in literature. Individual drugs that do not conform to generational naming are colored in black. **(C)** *h*-Woodward lactamome is colored using a heatmap with higher *h*-Woodward values in green and lower *h*-Woodward values in red. Bolded lactams marked with an asterisk indicate drugs with high allergy (>10%) relating to the R_1_ benzyl groups.

While generational labeling is a common method for describing β-lactams, particularly cephems (cephalosporins), this convention is not always consistent among researchers or pharmaceutical companies. Using consensus information from multiple literature sources, the relevant drugs in the Lactamome were assigned colors according to a generational scheme (Figure 5B). However, the generational Lactamome did not reveal any apparent trends or relationships that signaled generation. In most cases, terminal clusters are the same generation or differ by only one generation. In the case of cephems, later generations exhibit a broader spectrum of activity and less cross-reactivity with penicillin (Trubiano et al., 2017). The carbapenem class, while important for multidrug resistant infections, does not readily conform to chemical generation labeling. Similarly, β-lactamase inhibitors, mecillinam, and monobactams do not conform to multigenerational binning.

### Susceptibility to Hydrolysis

While factors like affinity, site accumulation, and pharmacokinetics affect *in vivo* efficacy, the minimum inhibitory concentrations (MICs) of antibiotics correlate with those having higher *h*-Woodward values. Correspondingly, higher *h*-Woodward value drugs are more prone to hydrolysis, exhibiting shorter aqueous and biological half-lives. For each compound examined, a self-consistent field calculation with geometry optimization was carried out using the modified neglect of diatomic overlap (MNDO) algorithm. The height of the trigonal pyramid with the lactam nitrogen at its apex and the nitrogen’s three bonded substituents at its base (i.e., *h*-Woodward) was measured for the optimized structure. However, the trend is not perfect, for example, clavulanate, while among the most reactive of the β–lactams, exhibits poor antibacterial activity against many organisms.

When the Lactamome was colored according to *h*-Woodward, an interesting pattern emerged (Figure 5C). Monobactams, except for tigemonam, exhibited the lowest *h* values, most of the cephems exhibited relatively low *h* values, and the first-through-third generation penams occupied the middle range of Woodward height. Carbapenems and the oxapenem, clavulanate, with its additional ring strain from the unsaturation at C2, demonstrated the highest *h*-Woodward values of all of the β-lactams analyzed herein. Using the Woodward parameter as a measure of reactivity may therefore serve as a guide to the relative ease of hydrolytic decomposition of β-lactams, with monobactams representing the least susceptible.

Because the β-lactams are minimally metabolized *in vivo*, their serum half-lives are largely determined through primary renal clearance. As exceptions, the ureido class (e.g., cefoperazone, piperacillin) undergo significant biliary clearance. Lactams that are more susceptible to hydrolysis tend to have greater activity, but also exhibit shorter biological half-lives. For example, carbapenems and penams tend toward lower plasma stability than cephems. Hydrolytic instability is one of the main reasons that many β-lactam antimicrobials need to be administered parenterally, reconstituted at the bedside or refrigerated, and dosed on a strict and frequent schedule. Compounds possessing unique pharmacokinetic properties not representative of their class may have markedly divergent half-lives. For example, ceftriaxone, ertapenem, and temocillin exhibit extensive protein binding (*t*_1/2_ > 6 h), and both cephalothin and cefotaxime are rapidly converted to their desacetyl metabolites (*t*_1/2_ < 1 h). An interesting recent development involving the fermentation isolation of clavulanate focused heavily on the minimization of its hydrolysis to improve its yield during manufacturing (Veeraraghavan et al., 2021).

While short serum half-lives may present challenges during administration of β-lactams, the increased rate of degradation *in vivo* may reduce the risk of occupational and environmental exposure after excretion. Such susceptibility to hydrolysis *ex vivo* can be exploited by chemical processes to decontaminate residues during manufacturing, compounding, and administration, as well as by waste-water treatment facilities to further reduce environmental hazards.

### Antibacterial Efficacy

**FIGURE 6:**
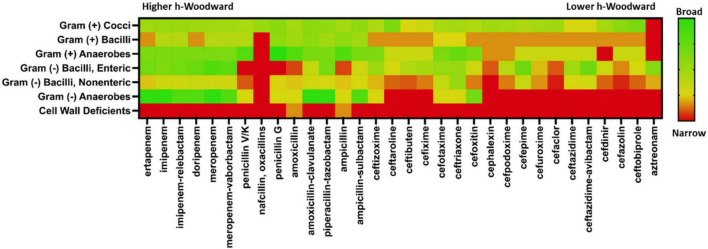
Antimicrobial spectra of β-lactams. Clinical activity data were taken from The Stanford Guide to Antimicrobial Therapy. A fractional coverage index was calculated using a weighted average of categorical coverage values reported. Heatmap color range is from green indicating high coverage and red indicating low coverage. Beta-lactams are ordered from highest *h*-Woodward value (ertapenem) to lowest (aztreonam).

While the optimal antibiotic strategy for patient care requires consideration of many factors, a primary criterion is the susceptibility of the infectious bacterial strain. Using The Sanford Guide to Antimicrobial Therapy (Gilbert et al., 2020), a “heat map” of susceptibility trends for β-lactam coverage by organism ultrastructure and metabolism was generated (Figure 6). When looking at the fractional activity against general classes of pathogenic bacteria, several specific patterns emerge. None of the β-lactams are particularly effective against cell wall-deficient microbes, which mostly include intracellular pathogens like mycoplasma and *Chlamydia trachomatis*. However, for infections typically caused by Gram-negative and Gram-positive bacteria, β-lactams present a myriad of options for good empiric or specific coverage. When comparing spectrum of activity versus *h*-Woodward values, a weak but correlative relationship is apparent. Beta-lactams with higher values generally exhibit broader spectrum of activity within and across groups of microbes than β-lactams with lower *h*-Woodward values. Notably, the monobactam aztreonam has the lowest *h*-Woodward value and a very narrow spectrum of activity. However, there are several exceptions, notably the relatively narrow spectrum of activity with nafcillin and oxacillin. As mentioned previously, this is due to the many factors affecting microbial killing, including site accumulation (particularly relevant in Gram-negative pathogens) and target affinity.

Good antimicrobial stewardship requires the use of an antimicrobial with the narrowest possible spectrum, but which still provides a high likelihood of infection clearance. For this reason, clinicians need to think about the most likely pathogens involved in specific infectious processes to guide an appropriately targeted empiric therapy. Targeted therapy also highlights the sustained need for rapid, cost-effective, and accurate diagnostics. As an example, absent other risk factors, cellulitis is generally caused by *S. pyogenes* or methicillin-susceptible *S. aureus* (MSSA). This condition can be treated effectively with nafcillin, any of the oxacillin subclass, or a first-generation cephalosporin like cefazolin. Cefazolin is also a safe alternative for patients with nafcillin hypersensitivity (Gandhi et al., 2021). Coverage should be expanded only with good reason, including colonization with methicillin-resistant *S. aureus* (MRSA), open wounds, or specific exposures (e.g., *Vibrio vulnificus* with saltwater exposure or *Aeromonas hydrophila* with freshwater exposure). One of the key deficiencies of the β-lactam class is its minimal activity against atypical and intracellular pathogens, as they are more resistant to alterations in peptidoglycan synthases. Non-lactam drugs–tetracyclines, macrolides, and fluoroquinolones–are likely more appropriate treatments for these microorganisms due to the variety of anti-infective mechanisms.

Special consideration should be given to infections with highly resistant organisms that have been verified with culture-based methods or nucleic acid amplification testing (NAAT). Conditions in which *Pseudomonas aeruginosa* is commonly implicated include burn wounds, healthcare-associated pneumonia, diabetic ulcers, and cystic fibrosis. For patients with these conditions, only a handful of β-lactam options exist, including ceftolozane-tazobactam, piperacillin-tazobactam, ceftazidime, cefepime, and carbapenems (except for ertapenem). Until the approval of ceftaroline, β-lactams were largely ineffective against MRSA; and were less commonly used than glycopeptide macrocyclic agents like vancomycin and daptomycin. Guidelines stipulate ceftaroline as a last-resort option for treatment of microbiologically proven cases of vancomycin-intermediate *S. aureus* (VISA, generally defined as vancomycin MIC ≥ 2 μg/mL) for which daptomycin cannot be used (e.g., lung infections). Ceftobiprole, though not yet approved in the United States, will likely occupy a similar niche.

Extended-spectrum β-lactamase (ESBL) producing strains of *E. coli* and *Klebsiella* spp. have become increasingly common in intra-abdominal infections as well as urinary tract infections. Carbapenems retain activity against these strains due to their remarkable β-lactamase stability, and newer agents employing diazabicyclooctane (DBO) β-lactamase inhibitors (e.g., ceftazidime-avibactam, imipenem-relebactam, and aztreonam-avibactam, which is still in development) also are effective. Carbapenemase-producing Gram-negative enterics also have emerged, for which combinations of carbapenems with DBOs and boronic acids (e.g., imipenem-relebactam and meropenem-vaborbactam) remain clinically efficacious. As with all infectious treatments the spectrum, dose, route of administration, and drug allergy should be carefully considered.

*Streptococcus pyogenes* (also known as or Group A *Streptococcus* or GAS) is the causative pathogen in several human diseases, including pharyngitis, scarlet fever, and necrotizing fasciitis. The susceptibility of this organism to β-lactams has remained remarkably consistent over many decades although there are recent indications that mutations in the gene encoding a PBP are widespread and can result in reduced susceptibility (Macris et al., 1998; Musser et al., 2020). However, given the historically consistent susceptibility, it was selected as a model organism to examine further the potential relationship between *h*-Woodward values and susceptibility to β-lactam antibiotics. The data were extracted from 95 different studies involving 10 strains of *S. pyogenes* (Wallmark, 1962; Adams, 1963; Sidell et al., 1963; Gravenkemper et al., 1965; Thornhill et al., 1969; Bergeron et al., 1973; Eykyn et al., 1973, 1976; Neu, 1974b,^1976^; Shibata et al., 1975; Ernst et al., 1976; Goto, 1977; Stewart and Bodey, 1977; Bodey and Le Blanc, 1978; Chabbert and Lutz, 1978; Verbist, 1978; Wise et al., 1978, 1980, 1988, 1991; Fass, 1979, 1990; Kamimura et al., 1979, 1984; Neu et al., 1979, 1981, 1984, 1989; Watanakunakorn and Glotzbecker, 1979; Weaver et al., 1979; Angehrn et al., 1980; Fu and Neu, 1980; Greenwood et al., 1980; Hinkle et al., 1980; Baker et al., 1981; Bodey et al., 1981, 1985; Cherubin et al., 1981; Eickhoff and Ehret, 1981; Istre et al., 1981; Jones et al., 1981, 1984; Stamm et al., 1981; Tsuchiya et al., 1981; Ahonkhai et al., 1982; Fainstein et al., 1982; Scully et al., 1983; Kessler et al., 1985; Toda et al., 1985; Une et al., 1985; Vuye and Pijck, 1985; Neu and Chin, 1986; Okamoto et al., 1987; Mine et al., 1988; Shelton and Nelson, 1988; Kayser et al., 1989; Yu and Neu, 1989; Arisawa et al., 1991; Chin et al., 1991, 1992; Emberton and Finlay, 1991; Limbert et al., 1991; Poch et al., 1992; Yan et al., 1993; Coonan and Kaplan, 1994; Tsuji et al., 1995, 1998; Suzuki et al., 1996; Cocuzza et al., 1997; Woodcock et al., 1997; Fontana et al., 1998; Kriebernegg et al., 1998; Sakagawa et al., 1998; Yamaguchi et al., 1998; Fuchs et al., 2001; Livermore et al., 2001; Pendland et al., 2002; Iizawa et al., 2004; Marchese et al., 2004; Lev et al., 2005; Stefani et al., 2008; Tempera et al., 2010; Camara et al., 2013; Fujimoto et al., 2013; Sakata, 2013; Yang et al., 2015; Yanik et al., 2015; Cotroneo et al., 2020; Jean et al., 2020; Musser et al., 2020; Ubukata et al., 2020; Yanagihara et al., 2020).

**FIGURE 7:**
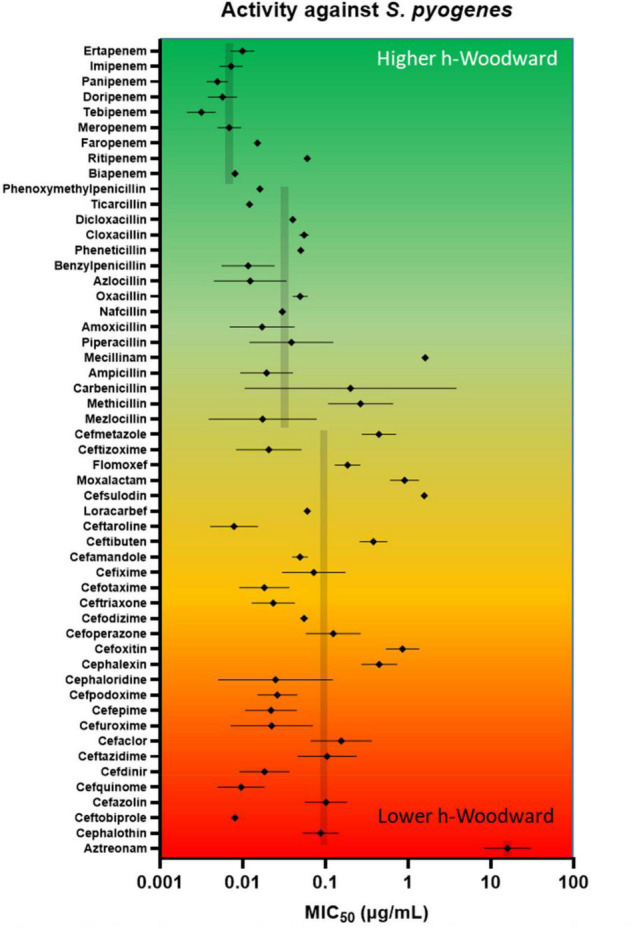
*In vitro* antimicrobial activity of lactams against *S. pyogenes*. A review of the literature on Gram-positive β-lactam activity was conducted in which susceptibility data were gleaned from 95 studies reporting data for at least 10 strains. MIC_50_ (presented as log values) were amassed, and geometric mean and geometric standard deviation were calculated. Geometric means for entire classes (e.g., penems) are shown in gray. Beta-lactams are ordered from highest *h*-Woodward value (ertapenem) to lowest (aztreonam).

As seen in the upper portion of Figure 7, β-lactams with higher *h*-Woodward values are the most potent options against *S. pyogenes*. Drugs with the lowest minimum inhibitory concentration values against 50% of the population (MIC_50_) also exhibit the lowest variation among different strains. Median susceptibility decreases (increased MIC_50_) at intermediate and higher *h*-Woodward values, although the overall trend suggests a parabolic relationship where drugs with intermediate *h*-Woodward values exhibit the lowest potency. Taken by class, penems are generally more biologically active than penams (0.007 vs. 0.024 μg/mL), and both exhibit greater efficacy than cephems (0.074 μg/ml). Aztreonam, the lone monobactam in use, was found to exhibit the lowest efficacy against *S. pyogenes* with a mean MIC_50_ of 16.5 μg/ml. The results of this analysis align with the previous observations related to the heat map, previously presented as Figure 6, that *h*-Woodward values impact antibacterial activity both within strains of *S. pyogenes* and across different groups of bacteria.

### Allergy and Other Immune Reactions

Hypersensitivity to β-lactams is among the most commonly reported drug allergies, representing 42.6% of all drug-induced anaphylaxis (Zagursky and Pichichero, 2018). However, prevalence is likely over-reported due to misinterpretation of mild drug reactions (e.g., diarrhea, mild drug rash, attribution of illness symptoms to drug). In fact, many individuals with reported β-lactam allergy can tolerate β-lactams due to this misattribution, and many of those with remote history of true allergic reaction are sometimes able to tolerate the drugs years later due to waning sensitivity (Romano et al., 2014). Even so, β-lactam allergy remains a clinically relevant problem, with skin testing generally yielding confirmed rates around 8% (3–10%) for penicillins and 1% (1–2%) for cephalosporins. Hypersensitivity to carbapenems and monobactams is rarer.

**FIGURE 8:**
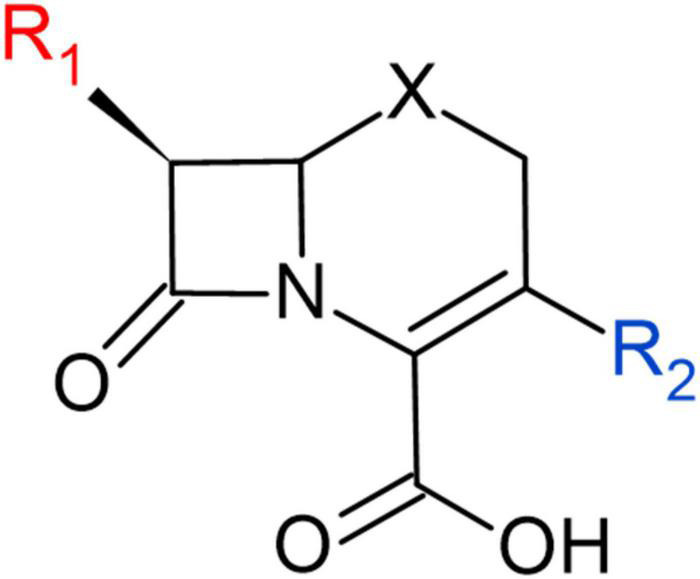
Structural representation of R_1_ and R_2_ chemical groups in cephem backbone.

Allergy is a result of immune activation in response to specific β-lactam substituents (Figure 1 and Supplementary Spreadsheet). The 2-azetidinone ring acts as a hapten, covalently modifying nucleophilic residues on host proteins to elicit an IgE response in individuals with specific human leukocyte antigen allotypes (Figure 8). While this covalently bound β-lactamoyl protein adduct is the major determinant of hypersensitivity, hydrolyzed small molecule products also can elicit some response.

Beta-lactam allergy is an immunologic type I hypersensitivity reaction, meaning that it occurs through mast cell degranulation in response to preformed IgE binding antigen recognition receptors (FcRε) on the mast cell surface. Subsequent release of histamine results in symptoms related to vasodilation (warmth and erythema), increased capillary permeability (edema), bronchoconstriction (dyspnea), and nociceptive stimulation (pain and pruritus). These symptoms are potentiated by the release of inflammatory eicosanoid derivatives, including prostaglandin D2, leukotriene B4, and leukotriene C4. In mild cases, this manifests as hives or wheezing, but in patients with high IgE titers, the reaction may proceed to anaphylaxis or anaphylactic shock. Because of the potential severity of the reaction, in addition to the poor accuracy of self-reporting mentioned above, identification of patients with true allergy through skin testing or measuring IgE against β-lactam products is critical to avoiding significant morbidity and mortality.

While not part of the group of IgE-mediated allergic syndrome, other significant immune reactions to the β-lactam class exist as well that are thought to occur through parallel mechanisms. Drug-induced immune hemolytic anemia (DIIHA) is a type II hypersensitivity reaction mediated by direct binding of IgG to erythrocytes, seen with relatively high frequency in β-lactam-treated subjects. One longitudinal study, documenting causes of DIIHA in Southern California over 30 years estimates that over 80% of cases are due to β-lactams (Garratty, 2009). Eosinophilic reactions are common as well, including drug-induced hypersensitivity syndrome (DiHS) with multiorgan involvement (previously known as drug reaction with eosinophilia and systemic symptoms or DRESS) caused by covalent modification of host proteins. Mucocutaneous eruption syndromes with epidermal detachment are also possible, arising from the activation of cytotoxic T-cells against keratinocytes.

There is a growing body of evidence suggesting the substituent adjacent to the lactam carbonyl (Figure 1), referred to as R_1_, is the primary driver of immunogenicity. Indeed, immunologic cross-reactivity is most often seen against structurally similar R_1_ groups of penams and cephems (i.e., ampicillin and cephalexin). One of the most prominent β-lactams associated with allergy is cefamandole, whose R_1_ is a hydroxybenzyl with a terminal aromatic. This structural relationship is not only common to such compounds as cephalexin (possessing an aminobenzyl moiety) but also to cephalothin, whose R_1_ aromatic is a thiophene, and cefuroxime whose R_1_ aromatic is a furan. These compounds, therefore, possess similar electronic and steric properties, or bioisosteric profiles. Such end group topology and electronic properties may play determinative roles in allergen character, possibly due to three-dimensional shape (“lock and key”) factors.

Compounds with 10% allergy response and containing similar aromatic R_1_ end groups have been bolded and tagged with asterisks in Figure 4. One careful study recommends skin reactivity testing for both penicillin and cephalosporin as 11% displayed positive skin test responses to the cephalosporin class, and 64% of cross-reactive patients responded negatively to cefamandole (Romano et al., 2004). In another study of 252 penicillin-allergic patients, ∼39% of patients treated with the cephalosporins cefaclor, cephalexin, cefadroxil, or cefamandole had an allergic response (Romano et al., 2018). Both studies show low cross-reactivity between penicillins and later-generation cephalosporins, such as ceftriaxone (∼3%). R_1_ groups containing phenyloxazole and piperazine also account for ∼3% of allergies. In cases where a patient may react in tests to dissimilar β-lactams, it is likely not due to true cross-reactivity, but rather to independent sensitivity to each drug. The low rate of cross-reactivity seen between penicillin and carbapenems (<1%), which possess a unique hydroxyethyl side chain, further supports the hypothesis that not only does the R_1_ bioisosterism play a dominant role, but aromatic end group chemistry appears causative (Romano et al., 2006, 2007; Atanaskovic-Markovic et al., 2008; Gaeta et al., 2015; Buonomo et al., 2016). Although not utilized on their own as an antibacterial therapy, β-lactam based β-lactam inhibitors also have been implicated in allergic reactions (Stover et al., 2019). The authors concluded that cross-allergenicity with β-lactams is likely with sulbactam and tazobactam. Cross-allergenicity is less likely with clavulanate, but still possible.

The involvement of the R_2_ side group in allergic response is the subject of greater uncertainty. While structural similarity of R_2_ has been considered as a possible factor, this group is frequently lost during ring opening, and any role it plays is generally quite small in comparison to the R_1_ side group. In the context of a β-lactam allergy, clinicians often look to different antimicrobial classes as alternatives. However, if the agent to which the patient has had an immune reaction is known, they may be able to use β-lactams of similar spectrum but differing chemical structures, both with regard to class and R_1_. For example, if a patient has had a severe reaction to amoxicillin in the past, cefpodoxime proxetil (a prodrug formulation) may be a suitable oral alternative due to its differently structured R_1_.

Similarly, clinicians often cite an allergy to penicillin G or amoxicillin as reason to avoid other penams, occasionally utilizing drugs of last defense when they might not be needed. As a clinical example, piperacillin-tazobactam is frequently avoided in the setting of a penicillin G allergy in favor of cefepime for similar Gram-negative enteric and pseudomonads coverage. Many clinicians, wary of cross-reactivity, will even avoid the cephalosporin class and prescribe a carbapenem. However, penicillin allergy may not preclude the use of piperacillin-tazobactam, as the benzyl functionality of penicillin G, to which the reaction likely occurred, is structurally very different from the R_1_ groups of cefepime or nitrogen-substituted R_1_ of piperacillin. For this reason, it is critical to keep accurate record of the β-lactam-based drugs to which the patient has reacted, as well as the type and severity of said reaction, so as to determine which other agents they are not likely to tolerate.

### Exposure and Environmental Concerns

Although β-lactam antibiotics do not meet the criteria for hazardous drugs established by the National Institute of Occupational Safety and Health (NIOSH), manufacturing and compounding of these drugs requires attention to reduce the risk of cross-contamination or occupational exposure to sensitive individuals. Current good manufacturing practice (cGMP) regulations require the use of methods to address cross-contamination [e.g., Title 21, Sections 211.42(d), 211.46(d), and 211.176 of the Code of Federal Regulations]. To further assist manufacturers, the FDA issued a draft guideline in 2013 entitled “Non-Penicillin Beta-Lactam Drugs: A cGMP Framework for Preventing Cross-Contamination” (Food and Drug Administration [FDA], 2013). Although most compounding pharmacies are 503a-type facilities and not required to operate under cGMP, the FDA often applies the described guidelines to pharmacy compounders. The guidelines are quite restrictive, including requiring segregation of “facilities” for “operations relating to the manufacture, processing, and packing” of β-lactam antibiotics. Additionally, an updated guidance document regarding insanitary conditions at compounding facilities was issued in November 2020 (Food and Drug Administration [FDA], 2020). It lists several examples of insanitary conditions applicable to the production of sterile and non-sterile drug, including: “Processing of β-lactams without complete and comprehensive separation from non-β-lactam products.” While segregation may not be absolutely required (or feasible) for all compounding activities, it is important to reduce the risk of cross-contamination through effective decontamination of surfaces and equipment after manipulations involving β-lactam antibiotics.

Another concern involves the environmental persistence of antibiotics, including β-lactams, in the environment. Antibiotics used to treat humans and livestock can escape into the environment by several routes, including improper disposal and excretion through urine and feces (Kivits et al., 2018; Szekeres et al., 2018). Conventional waste-water treatment processes are not effective at removing or destroying these drugs (Song et al., 2019). Persistence of these drugs in the environment can therefore result in selective pressures on environmental bacteria, leading to development of resistance genes which then may be transferred to human or animal pathogens (Bengtsson-Palme et al., 2018). β-lactams with intermediate or high *h*-Woodward values are more susceptible to decontamination *via* alkaline or acid hydrolysis at the 2-azetidinone ring (Lang et al., 1978). The kinetics of inactivation by such processes are related to the extent of ring activation. Thus, cephems tend to be more resistant to degradation, followed by penams, while carbapenems are the most susceptible.

While further studies are needed, there is evidence that oxidizing agents may decontaminate β-lactam antibiotics (Lorcheim, 2011; He et al., 2014; Zhang et al., 2017). These chemicals commonly are used for disinfection of surfaces contaminated with β-lactam antibiotics as well as in waste-water treatment systems. Additional research should establish how oxidizers such as peracetic acid, hydrogen peroxide, and hypochlorous acid would perform as decontamination strategies within the four major classes of β-lactam antibiotics, and whether such oxidants would differ in efficacy by pH and h-Woodward.

## Conclusion

β-lactams remain an important and highly utilized class of antibiotics. In the outpatient setting, amoxicillin and cephalexin are consistently among the most prescribed drugs in the United States, and ceftriaxone and piperacillin-tazobactam are staples of empiric inpatient treatment. Over their decades of prominence, however, concerns have arisen among clinicians and laboratories regarding their efficacy and environmental persistence. Among these concerns are serious allergic responses and acquisition of drug resistance by environmental bacteria.

While non-β-lactam antibiotics have gained increased attention, the utility of β-lactam antibiotics remains important, not only in the human clinical situation but also in a wide array of veterinary uses. The current 5th generation β-lactam are undergoing clinical trials and new β-lactams are expected to see less development, as non-β-lactam antibiotics, and combinations of β-lactams with lactamase inhibitors take center stage (Figure 5B). The current work structurally classified the β-lactam class of antimicrobials to aid clinical decision making, as well as inform patient, worker, and environmental safety. We have organized our structural analysis into an easily visualized tool, the Lactamome (Figure 4), which can be colorized according to several chemical properties, including overall molecular shape (Figures 3, 5A) and propensity for reaction with nucleophiles, i.e., *h*-Woodward (Figure 3). It should also be noted that when colored by allergic potential, one would obtain a chemogram strongly resembling Figure 5C, depicting h-Woodward.

The *h*-Woodward colored Lactamome (Figure 5C) indicates that higher values for penems and penams (Figures 2, 5) would be the most susceptible to chemically catalyzed degradation. This greater propensity for hydrolysis is important in reducing occupational exposures, especially given that the highest rate of reported β-lactam hypersensitivity is to the penam class. Logically, more stringent cleaning is needed if a more allergy-prone lactam is used, but fortunately, the penams’ high *h*-Woodward values indicate decontamination protocols involving hydrolysis should be efficient. In the case of cephems, while their overall rate of allergenicity is somewhat lower than that of penams, many first-generation agents from this subclass have high structural similarity to the first-generation penams (e.g., penicillin and cephalexin) and are, therefore, more likely to be allergens (Figure 5B). Therefore, care should be taken in the decontamination of surfaces or areas exposed to those cephems with a higher possibility for cross-reactivity.

Antibacterial efficacy also appears to trend with *h*-Woodward values. Drugs with higher h-Woodward values tend to have lower MICs against *S. pyogenes* and activity against a broader spectrum of bacteria (Figure 7). However, given the multitude of mechanisms involved with susceptibility across different species and types of bacteria, it is not clear if *h*-Woodward values would be a reliable criterion for matching β-lactams with particular pathogens (Figure 6). It is apparent that the “generational” nomenclature is a function of product development over time and generally is not well correlated with molecular structure or activity.

Unlike the correlation of *h*-Woodward values with both high intrinsic potency and high hydrolysis potential, allergenicity is impacted by additional factors, including the position of the R_1_ group (Figure 8). In terms of selecting alternate β-lactams due to allergy, the Lactamome, when used in combination with the spectrum data (Figure 6) provides information for treatment options. These analyses also may guide future studies of efficacy to broaden recommended uses of known β-lactams. For example, if a patient with spontaneous bacterial peritonitis has a documented anaphylactic response to empiric cefotaxime, they are likely to have an adverse reaction to structurally similar ceftriaxone as well. In these situations, guidelines suggest a fluoroquinolone as the next suitable option. Using the structure and spectrum data presented, however, one might consider ampicillin-sulbactam, a decision for which positive data exists but a full-scale prospective non-inferiority trial has not been conducted (Guo et al., 2019).

The structural characterization presented in this study may provide a helpful guide to selecting antibiotic agents, especially for persons having certain allergic response histories, as well as to inform proper decontamination methods. The Lactamome may assist clinicians in assessing structural similarity and therefore help recognize, relatively speaking, which drug would be most effective, but exhibit lower risks of clinical cross-sensitivity. By first grouping the β-lactam antibiotics and then using coloring schemes, it is possible to quickly probe the entire genus. The Lactamome should also be useful in alerting practitioners to potential workplace hazards and assist in the development of decontamination strategies to reduce the risk of cross-contamination, occupational exposure, and environmental persistence.

## Author Contributions

All authors conceived and wrote the manuscript together. JT generated the spectrum and activity graphs. YP and AM generated the lactamome chemograms.

## Conflict of Interest

This study received funding from Contec, Inc. The funder Contec, Inc. had the following involvement with the study: LP and MW helped conceive of the analysis, aided in analysis, co-wrote the manuscript, helped with graphical design, and aided in revisions. LP and MW were employed by Contec, Inc. MB was employed by Intramed Plus. The remaining authors declare that the research was conducted in the absence of any commercial or financial relationships that could be construed as a potential conflict of interest.

## Publisher’s Note

All claims expressed in this article are solely those of the authors and do not necessarily represent those of their affiliated organizations, or those of the publisher, the editors and the reviewers. Any product that may be evaluated in this article, or claim that may be made by its manufacturer, is not guaranteed or endorsed by the publisher.

## References

[B1] Abrahamsson S. Hodgkin D. C. Maslen E. N. (1963). The crystal structure of phenoxymethylpenicillin. *Biochem. J.* 86 514–535. 10.1042/bj0860514 14010750 PMC1201786

[B2] Adams C. W. (1963). Multiple factors in the pathogenesis of atherosclerosis. *Guys Hosp. Rep.* 112 222–253. 14043898

[B3] Ahonkhai V. I. Cherubin C. E. Shulman M. A. (1982). In vitro activity of cefodizime (HR-221). *Antimicrob. Agents Chemother.* 22 715–718. 10.1128/aac.22.4.715 6295264 PMC183824

[B4] Aldridge S. Parascandola J. Sturchio J. L. American Chemical Society, and Royal Society of Chemistry (1999). *The Discovery and Development of Penicillin 1928-1945.* Washington, DC: American Chemical Society.

[B5] Angehrn P. Probst P. J. Reiner R. Then R. L. (1980). Ro 13-9904, a long-acting broad-spectrum cephalosporin: in vitro and in vivo studies. *Antimicrob. Agents Chemother.* 18 913–921. 10.1128/aac.18.6.913 6972194 PMC352988

[B6] Aoki T. Yoshizawa H. Yamawaki K. Yokoo K. Sato J. Hisakawa S. (2018). Cefiderocol (S-649266), a new siderophore cephalosporin exhibiting potent activities against *Pseudomonas aeruginosa* and other gram-negative pathogens including multi-drug resistant bacteria: structure activity relationship. *Eur. J. Med. Chem.* 155 847–868. 10.1016/j.ejmech.2018.06.014 29960205

[B7] Arisawa M. Sekine Y. Shimizu S. Takano H. Angehrn P. Then R. L. (1991). In vitro and in vivo evaluation of Ro 09-1428, a new parenteral cephalosporin with high antipseudomonal activity. *Antimicrob. Agents Chemother.* 35 653–659. 10.1128/aac.35.4.653 1906261 PMC245074

[B8] Asai M. Haibara K. Muroi M. Kintaka K. Kishi T. (1981). Sulfazecin, a novel beta-lactam antibiotic of bacterial origin. Isolation and chemical characterization. *J. Antibiot.* 34 621–627. 10.7164/antibiotics.34.621 7024230

[B9] Atanaskovic-Markovic M. Gaeta F. Medjo B. Viola M. Nestorovic B. Romano A. (2008). Tolerability of meropenem in children with IgE-mediated hypersensitivity to penicillins. *Allergy* 63 237–240. 10.1111/j.1398-9995.2007.01532.x 18186815

[B10] Backman T. W. Cao Y. Girke T. (2011). ChemMine tools: an online service for analyzing and clustering small molecules. *Nucleic Acids Res.* 39 W486–W491. 10.1093/nar/gkr320 21576229 PMC3125754

[B11] Baker C. N. Thornsberry C. Facklam R. R. (1981). Synergism, killing kinetics, and antimicrobial susceptibility of group A and B streptococci. *Antimicrob. Agents Chemother.* 19 716–725. 10.1128/aac.19.5.716 7027921 PMC181512

[B12] Basker M. J. Boon R. J. Hunter P. A. (1980). Comparative antibacterial properties in vitro of seven olivanic acid derivatives: MM 4550, MM 13902, MM 17880, MM 22380, MM 22381, MM 22382 and MM 22383. *J. Antibiot.* 33 878–884. 10.7164/antibiotics.33.878 6968745

[B13] Bassetti M. Giacobbe D. R. Castaldo N. Russo A. Vena A. (2021). Role of new antibiotics in extended-spectrum beta- lactamase-, AmpC- infections. *Curr. Opin. Infect. Dis.* 34 748–755. 10.1097/QCO.0000000000000789 34581282

[B14] Batchelder H. R. Story-Roller E. Lloyd E. P. Kaushik A. Bigelow K. M. Maggioncalda E. C. (2020). Development of a penem antibiotic against *Mycobacteroides abscessus*. *Commun. Biol.* 3:741. 10.1038/s42003-020-01475-2 33288821 PMC7721803

[B15] Bengtsson-Palme J. Kristiansson E. Larsson D. G. J. (2018). Environmental factors influencing the development and spread of antibiotic resistance. *FEMS Microbiol. Rev.* 42:fux053. 10.1093/femsre/fux053 29069382 PMC5812547

[B16] Bergeron M. G. Brusch J. L. Barza M. Weinstein L. (1973). Bactericidal activity and pharmacology of cefazolin. *Antimicrob. Agents Chemother.* 4 396–401. 10.1128/aac.4.4.396 4598612 PMC444566

[B17] Bhatnagar A. Ransom E. M. Machado M. J. Boyd S. Reese N. Anderson K. (2021). Assessing the *in vitro* impact of ceftazidime on aztreonam/avibactam susceptibility testing for highly resistant MBL-producing Enterobacterales. *J. Antimicrob. Chemother.* 76 979–983. 10.1093/jac/dkaa531 33367916 PMC10838604

[B18] Bodey G. P. Fainstein V. Hinkle A. M. (1981). Comparative in vitro study in new cephalosporins. *Antimicrob. Agents Chemother.* 20 226–230. 10.1128/aac.20.2.226 7283419 PMC181667

[B19] Bodey G. P. Ho D. H. LeBlanc B. (1985). In vitro studies of BMY-28142, a new broad-spectrum cephalosporin. *Antimicrob. Agents Chemother.* 27 265–269. 10.1128/aac.27.2.265 3838637 PMC176251

[B20] Bodey G. P. Le Blanc B. (1978). Piperacillin: in vitro evaluation. *Antimicrob. Agents Chemother.* 14 78–87. 10.1128/aac.14.1.78 28694 PMC352408

[B21] Boyd D. B. (1984). Electronic structures of cephalosporins and penicillins. 15. Inductive effect of the 3-position side chain in cephalosporins. *J. Med. Chem.* 27 63–66. 10.1021/jm00367a012 6690684

[B22] Boyd D. B. Hermann R. B. Presti D. E. Marsh M. M. (1975). Electronic structures of cephalosporins and penicillins. 4. Modeling acylation by the beta-lactam ring. *J. Med. Chem.* 18 408–417. 10.1021/jm00238a018 1121009

[B23] Buonomo A. Pascolini L. Rizzi A. Aruanno A. Pecora V. Ricci A. G. (2016). Cross-reactivity and tolerability of ertapenem in patients with IgE-mediated hypersensitivity to beta-lactams. *J. Investig. Allergol. Clin. Immunol.* 26 100–105. 10.18176/jiaci.0019 27164625

[B24] Bush K. Bradford P. A. (2016). beta-lactams and beta-lactamase inhibitors: an overview. *Cold Spring Harb. Perspect. Med.* 6:a025247. 10.1101/cshperspect.a025247 27329032 PMC4968164

[B25] Butler M. S. Gigante V. Sati H. Paulin S. Al-Sulaiman L. Rex J. H. (2022). Analysis of the clinical pipeline of treatments for drug resistant bacterial infections: despite progress, more action is needed. *Antimicrob. Agents Chemother.* 10.1128/AAC.01991-21 [Online ahead of print]. 35007139 PMC8923189

[B26] Camara M. Dieng A. Boye C. S. (2013). Antibiotic susceptibility of *Streptococcus pyogenes* isolated from respiratory tract infections in dakar, senegal. *Microbiol. Insights* 6 71–75. 10.4137/MBI.S12996 24826076 PMC3987753

[B27] Cazzola M. Brancaccio V. De Giglio C. Paterno E. Matera M. G. Rossi F. (1993). Flomoxef, a new oxacephem antibiotic, does not cause hemostatic defects. *Int. J. Clin. Pharmacol. Ther. Toxicol.* 31 148–152. 8468113

[B28] Chabbert Y. A. Lutz A. J. (1978). HR 756, the syn isomer of a new methoxyimino cephalosporin with unusual antibacterial activity. *Antimicrob. Agents Chemother.* 14 749–754. 10.1128/aac.14.5.749 31836 PMC352545

[B29] Cherubin C. E. Corrado M. L. Sierra M. F. Gombert M. E. Shulman M. (1981). Susceptibility of gram-positive cocci to various antibiotics, including cefotaxime, moxalactam, and N-formimidoyl thienamycin. *Antimicrob. Agents Chemother.* 20 553–555. 10.1128/aac.20.4.553 6282200 PMC181744

[B30] Chin N. X. Gu J. W. Fang W. Neu H. C. (1991). In vitro activity and beta-lactamase stability of GR69153, a new long-acting cephalosporin. *Antimicrob. Agents Chemother.* 35 259–266. 10.1128/aac.35.2.259 2024959 PMC244988

[B31] Chin N. X. Gu J. W. Fang W. Neu H. C. (1992). In vitro activity of cefquinome, a new cephalosporin, compared with other cephalosporin antibiotics. *Diagn. Microbiol. Infect. Dis.* 15 331–337. 10.1016/0732-8893(92)90019-p 1611848

[B32] Cocuzza C. Blandino G. Mattina R. Nicoletti F. Nicoletti G. (1997). Antibiotic susceptibility of group a streptococci in 2 Italian cities: Milano and Catania. *Microb. Drug Resist.* 3 379–384. 10.1089/mdr.1997.3.379 9442491

[B33] Coonan K. M. Kaplan E. L. (1994). In vitro susceptibility of recent North American group a streptococcal isolates to eleven oral antibiotics. *Pediatr. Infect. Dis. J.* 13 630–635. 10.1097/00006454-199407000-00009 7970952

[B34] Cotroneo N. Rubio A. Critchley I. A. Pillar C. Pucci M. J. (2020). *In Vitro* and *In Vivo* characterization of tebipenem, an oral carbapenem. *Antimicrob. Agents Chemother.* 64:e02240-19. 10.1128/AAC.02240-19 32423950 PMC7526814

[B35] Cunha B. A. (1993). Aztreonam. *Urology* 41 249–258. 10.1016/0090-4295(93)90568-u 8442309

[B36] Decuyper L. Jukic M. Sosic I. Zula A. D’Hooghe M. Gobec S. (2018). Antibacterial and beta-lactamase inhibitory activity of monocyclic beta-lactams. *Med. Res. Rev.* 38 426–503. 10.1002/med.21443 28815732

[B37] Dexter D. D. van der Veen J. M. (1978). Conformations of penicillin G: crystal structure of procaine penicillin G monohydrate and a refinement of the structure of potassium penicillin G. *J. Chem. Soc. Perkin* 1 185–190. 10.1039/p19780000185 565366

[B38] Diaz N. Suarez D. Sordo T. L. Tunon I. Silla E. (2002). Water-assisted alkaline hydrolysis of monobactams: a theoretical study. *Chemistry* 8 859–867. 11857700 10.1002/1521-3765(20020215)8:4<859::aid-chem859>3.0.co;2-i

[B39] Dobias J. Denervaud-Tendon V. Poirel L. Nordmann P. (2017). Activity of the novel siderophore cephalosporin cefiderocol against multidrug-resistant Gram-negative pathogens. *Eur. J. Clin. Microbiol. Infect. Dis.* 36 2319–2327. 10.1007/s10096-017-3063-z 28748397

[B40] Eagle H. (1946). The relative activity of penicillins F, G, K, and X against spirochetes and streptococci in vitro. *J. Bacteriol.* 52 81–88. 10.1128/JB.52.1.81-88.1946 20994872

[B41] Eagle H. Technical Assistance of Arlyne D. Musselman (1947). The therapeutic activity of penicillins F, G, K, and X in experimental infections with pneumococcus type I and *Streptococcus pyogenes*. *J. Exp. Med.* 85 175–186. 10.1084/jem.85.2.175 19871606 PMC2135693

[B42] Eickhoff T. C. Ehret J. (1981). Comparative in vitro studies of Ro 13-9904, a new cephalosporin derivative. *Antimicrob. Agents Chemother.* 19 435–442. 10.1128/aac.19.3.435 6264845 PMC181450

[B43] Emberton P. Finlay D. B. (1991). Normal roentgen variant simulating disease: coronal suture overlying maxilla simulating fracture. *Clin. Radiol.* 43:217. 10.1016/s0009-9260(05)80485-4 2013201

[B44] Entenza J. M. Hohl P. Heinze-Krauss I. Glauser M. P. Moreillon P. (2002). BAL9141, a novel extended-spectrum cephalosporin active against methicillin-resistant *Staphylococcus aureus* in treatment of experimental endocarditis. *Antimicrob. Agents Chemother.* 46 171–177. 10.1128/aac.46.1.171-177.2002 11751129 PMC126986

[B45] Ernest I. Gosteli J. Greengrass C. W. Holick W. Woodward R. B. (1978). The penems, a new class of beta-lactam antibiotics: 6-acylaminopenem-3-carboxylic acids. *J. Am. Chem. Soc.* 100 8214–8222. 10.1021/ja00494a032

[B46] Ernst E. C. Berger S. Barza M. Jacobus N. V. Tally F. P. (1976). Activity of cefamandole and other cephalosporins against aerobic and anaerobic bacteria. *Antimicrob. Agents Chemother.* 9 852–855. 10.1128/aac.9.5.852 949182 PMC429632

[B47] Eykyn S. Jenkins C. King A. Phillips I. (1973). Antibacterial activity of cefamandole, a new cephalosporin antibiotic, compared with that of cephaloridine, cephalothin, and cephalexin. *Antimicrob. Agents Chemother.* 3 657–661. 10.1128/aac.3.6.657 4790616 PMC444475

[B48] Eykyn S. Jenkins C. King A. Phillips I. (1976). Antibacterial activity of cefuroxime, a new cephalosporin antibiotic, compared with that of cephaloridine, cephalothin, and cefamandole. *Antimicrob. Agents Chemother.* 9 690–695. 10.1128/aac.9.4.690 1267441 PMC429599

[B49] Fainstein V. Weaver S. Bodey G. P. (1982). Comparative in vitro study of SQ26, 776. *Antimicrob. Agents Chemother.* 21 294–298. 10.1128/aac.21.2.294 6918206 PMC181876

[B50] Fass R. J. (1979). In vitro activity of LY127935. *Antimicrob. Agents Chemother.* 16 503–509. 10.1128/aac.16.4.503 518080 PMC352889

[B51] Fass R. J. (1990). In vitro activity of BAY v 3522, a new oral cephalosporin. *Antimicrob. Agents Chemother.* 34 1855–1857. 10.1128/aac.34.9.1855 2126697 PMC171948

[B52] Fleming A. (2001). On the antibacterial action of cultures of a penicillium, with special reference to their use in the isolation of B. influenzae. 1929. *Bull. World Health Organ.* 79 780–790. 11545337 PMC2566493

[B53] Fontana R. Altamura M. Arcamone F. Mazzariol A. Morandotti G. Sperning R. (1998). MEN 10700, a new penem antibiotic: in-vitro activity and its correlation with beta-lactamase stability, PBP affinity and diffusion through the bacterial cell wall. *J. Antimicrob. Chemother.* 41 513–525. 10.1093/jac/41.5.513 9630405

[B54] Food and Drug Administration [FDA] (2013). *Non-Penicillin Beta-Lactam Drugs: A CGMP Framework for Preventing Cross-Contamination*, ed. CDER (Silver Spring, MD: Food and Drug Administration).

[B55] Food and Drug Administration [FDA] (2020). *Insanitary Conditions at Compounding Facilities - Guidance for Industry*, ed. CDER (Silver Spring, MD: Food Drug Administration).

[B56] Foye W. O. Lemke T. L. Williams D. A. (2008). *Foye’s Principles of Medicinal Chemistry.* Philadelphia, PA: Lippincott Williams & Wilkins.

[B57] Frere J. M. Ghuysen J. M. Iwatsubo M. (1975). Kinetics of interaction between the exocellular DD-carboxypeptidase-transpeptidase from Streptomyces R61 and beta-lactam antibiotics. A choice of models. *Eur. J. Biochem.* 57 343–351. 10.1111/j.1432-1033.1975.tb02307.x 1175647

[B58] Fu K. P. Neu H. C. (1980). Antibacterial activity of ceftizoxime, a beta-lactamase-stable cephalosporin. *Antimicrob. Agents Chemother.* 17 583–590. 10.1128/aac.17.4.583 6967294 PMC283835

[B59] Fuchs P. C. Barry A. L. Brown S. D. (2001). In vitro activities of ertapenem (MK-0826) against clinical bacterial isolates from 11 North American medical centers. *Antimicrob. Agents Chemother.* 45 1915–1918. 10.1128/AAC.45.6.1915-1918.2001 11353653 PMC90573

[B60] Fujimoto K. Takemoto K. Hatano K. Nakai T. Terashita S. Matsumoto M. (2013). Novel carbapenem antibiotics for parenteral and oral applications: in vitro and in vivo activities of 2-aryl carbapenems and their pharmacokinetics in laboratory animals. *Antimicrob. Agents Chemother.* 57 697–707. 10.1128/AAC.01051-12 23147735 PMC3553697

[B61] Gaeta F. Valluzzi R. L. Alonzi C. Maggioletti M. Caruso C. Romano A. (2015). Tolerability of aztreonam and carbapenems in patients with IgE-mediated hypersensitivity to penicillins. *J. Allergy Clin. Immunol.* 135 972–976. 10.1016/j.jaci.2014.10.011 25457154

[B62] Gandhi A. M. Shah M. D. Donohue L. E. Cox H. L. Eby J. C. (2021). Tolerability of cefazolin in nafcillin-intolerant patients for the treatment of methicillin-susceptible *Staphylococcus aureus* infections. *Clin. Infect. Dis.* 73 1650–1655. 10.1093/cid/ciab368 33905485 PMC8825210

[B63] Garratty G. (2009). Drug-induced immune hemolytic anemia. *Hematology Am. Soc. Hematol. Educ. Program* 2009 73–79. 10.1182/asheducation-2009.1.73 20008184

[B64] Georgopapadakou N. H. Smith S. A. Sykes R. B. (1982). Mode of action of azthreonam. *Antimicrob. Agents Chemother.* 21 950–956. 10.1128/aac.21.6.950 6180685 PMC182051

[B65] Gilbert D. N. Chambers H. F. Saag M. S. Pavia A. T. Black D. Boucher H. W. (2020). *The Sanford Guide to Antimicrobial Therapy 2020.* Sperryville, VA: Antimicrobial Therapy, Inc.

[B66] Glover S. A. Rosser A. A. (2012). Reliable determination of amidicity in acyclic amides and lactams. *J. Org. Chem.* 77 5492–5502. 10.1021/jo300347k 22646836

[B67] Godzeski C. W. Brier G. Pavey D. E. (1963). Cephalothin, a new cephalosporin with a broad antibacterial spectrum. I. In vitro studies employing the gradient plate technique. *Appl. Microbiol.* 11 122–127. 13963283 10.1128/am.11.2.122-127.1963 PMC1057954

[B68] Goffin C. Ghuysen J. M. (1998). Multimodular penicillin-binding proteins: an enigmatic family of orthologs and paralogs. *Microbiol. Mol. Biol. Rev.* 62 1079–1093. 9841666 10.1128/mmbr.62.4.1079-1093.1998 PMC98940

[B69] Goldberg J. A. Kumar V. Spencer E. J. Hoyer D. Marshall S. H. Hujer A. M. (2021). A gamma-lactam siderophore antibiotic effective against multidrug-resistant *Pseudomonas aeruginosa*, *Klebsiella pneumoniae*, and *Acinetobacter* spp. *Eur. J. Med. Chem.* 220:113436. 10.1016/j.ejmech.2021.113436 33933754 PMC11444265

[B70] Goto S. (1977). The *in vitro* and *in vivo* antibacterial activity of cefuroxime. *Proc. R. Soc. Med.* 70 (Suppl. 9), 56–62. 10.1177/00359157770700S911 PMC1543250 20919419

[B71] Gravenkemper C. F. Bennett J. V. Brodie J. L. Kirby W. M. (1965). Dicloxacillin. In Vitro and pharmacologic comparisons with Oxacillin and Cloxacillin. *Arch. Intern. Med.* 116 340–345. 10.1001/archinte.1965.03870030020005 14325906

[B72] Graves-Woodward K. Pratt R. F. (1998). Reaction of soluble penicillin-binding protein 2a of methicillin-resistant *Staphylococcus aureus* with beta-lactams and acyclic substrates: kinetics in homogeneous solution. *Biochem. J.* 332(Pt 3), 755–761. 10.1042/bj3320755 9620879 PMC1219537

[B73] Green G. F. Page J. E. Staniforth S. E. (1965). Cephalosporanic acids. I. Infrared absorption and proton magnetic resonance spectra of cephalosporin and penicillin analogues. *J. Chem. Soc.* 65 1595–1605. 10.1039/jr9650001595 14288320

[B74] Greenwood D. (2008). *Antimicrobial Drugs: Chronicle of a Twentieth Century Medical Triumph.* Oxford: Oxford University Press.

[B75] Greenwood D. Pearson N. Eley A. O’Grady F. (1980). Comparative in vitro activities of cefotaxime and ceftizoxime (FK749): new cephalosporins with exceptional potency. *Antimicrob. Agents Chemother.* 17 397–401. 10.1128/aac.17.3.397 6252829 PMC283798

[B76] Guo J. Shi J. Wang H. Chen H. Liu S. Li J. (2019). Emerging Gram-positive bacteria and drug resistance in cirrhosis patients with spontaneous bacterial peritonitis: a retrospective study. *Exp. Ther. Med.* 17 4568–4576. 10.3892/etm.2019.7502 31186679 PMC6507503

[B77] Hamilton-Miller J. M. Kerry D. W. Brumfitt W. (1974). An in vivo comparison of cefoxitin, a semi-synthetic cephamycin, with cephalothin. *J. Antibiot.* 27 42–48. 10.7164/antibiotics.27.42 4843049

[B78] Hancock R. E. Bellido F. (1992). Factors involved in the enhanced efficacy against gram-negative bacteria of fourth generation cephalosporins. *J. Antimicrob. Chemother.* 29 (Suppl. A), 1–6. 10.1093/jac/29.suppl_a.1 1601751

[B79] He X. Mezyk S. P. Michael I. Fatta-Kassinos D. Dionysiou D. D. (2014). Degradation kinetics and mechanism of beta-lactam antibiotics by the activation of H2O2 and Na2S2O8 under UV-254nm irradiation. *J. Hazard. Mater.* 279 375–383. 10.1016/j.jhazmat.2014.07.008 25086235

[B80] Hinkle A. M. LeBlanc B. M. Bodey G. P. (1980). In vitro evaluation of cefoperazone. *Antimicrob. Agents Chemother.* 17 423–427. 10.1128/aac.17.3.423 6448578 PMC283803

[B81] Hsieh W. C. Ho S. W. (1975). Evaluation of antibacterial activities of cephalosporin antibiotics: cefazolin, cephaloridine, cephalothin, and cephalexin. *Zhonghua Min. Guo Wei Sheng Wu Xue Za Zhi* 8 1–11. 1097210

[B82] Hughes D. L. (2017a). Patent review of manufacturing routes to fifth-generation cephalosporin drugs. Part 1, ceftolozane. *Org. Process Res. Dev.* 21 430–443. 10.1021/acs.oprd.7b00033

[B83] Hughes D. L. (2017b). Patent review of manufacturing routes to fifth-generation cephalosporin drugs. Part 2, ceftaroline fosamil and ceftobiprole medocaril. *Org. Process Res. Dev.* 21 800–815. 10.1021/acs.oprd.7b00143

[B84] Hujer A. M. Kania M. Gerken T. Anderson V. E. Buynak J. D. Ge X. (2005). Structure-activity relationships of different beta-lactam antibiotics against a soluble form of *Enterococcus faecium* PBP5, a type II bacterial transpeptidase. *Antimicrob. Agents Chemother.* 49 612–618. 10.1128/AAC.49.2.612-618.2005 15673741 PMC547200

[B85] Huson D. H. Richter D. C. Rausch C. Dezulian T. Franz M. Rupp R. (2007). Dendroscope: an interactive viewer for large phylogenetic trees. *BMC Bioinformatics* 8:460. 10.1186/1471-2105-8-460 18034891 PMC2216043

[B86] Iizawa Y. Nagai J. Ishikawa T. Hashiguchi S. Nakao M. Miyake A. (2004). In vitro antimicrobial activity of T-91825, a novel anti-MRSA cephalosporin, and in vivo anti-MRSA activity of its prodrug, TAK-599. *J. Infect. Chemother.* 10 146–156. 10.1007/s10156-004-0309-3 15290453

[B87] Imada A. Kitano K. Kintaka K. Muroi M. Asai M. (1981). Sulfazecin and isosulfazecin, novel beta-lactam antibiotics of bacterial origin. *Nature* 289 590–591. 10.1038/289590a0 7007891

[B88] Inamoto Y. Chiba T. Kamimura T. Takaya T. (1988). FK 482, a new orally active cephalosporin synthesis and biological properties. *J. Antibiot.* 41 828–830. 10.7164/antibiotics.41.828 3255303

[B89] Inamoto Y. Chiba T. Sakane K. Kamimura T. Takaya T. (1990a). [Studies on FK482. II. Synthesis and structure-activity relationships of 7 beta-[(Z)-2-(2-aminothiazol-4-yl)-2-substituted acetamido]-3-vinyl-3-cephem-4-carboxylic acid derivatives]. *Yakugaku Zasshi* 110 246–257. 10.1248/yakushi1947.110.4_246 2376819

[B90] Inamoto Y. Sakane K. Kamimura T. Takaya T. (1990b). [Studies on FK482 (Cefdinir). III. Synthesis and structure-activity relationships of 7 beta-[(Z)-2-aryl-2-hydroxyiminoacetamido]-3-vinyl-3- cephem-4-carboxylic acid derivatives]. *Yakugaku Zasshi* 110 658–664. 10.1248/yakushi1947.110.9_658 2262878

[B91] Ishikawa T. Matsunaga N. Tawada H. Kuroda N. Nakayama Y. Ishibashi Y. (2003). TAK-599, a novel N-phosphono type prodrug of anti-MRSA cephalosporin T-91825: synthesis, physicochemical and pharmacological properties. *Bioorg. Med. Chem.* 11 2427–2437. 10.1016/s0968-0896(03)00126-3 12735989

[B92] Ismalaj E. (2022). *Penicillins.* Oxford: Elsevier.

[B93] Issa N. C. Rouse M. S. Piper K. E. Wilson W. R. Steckelberg J. M. Patel R. (2004). In vitro activity of BAL9141 against clinical isolates of gram-negative bacteria. *Diagn. Microbiol. Infect. Dis.* 48 73–75. 10.1016/j.diagmicrobio.2003.09.006 14761726

[B94] Istre G. R. Welch D. F. Marks M. I. Moyer N. (1981). Susceptibility of group A beta-hemolytic Streptococcus isolates to penicillin and erythromycin. *Antimicrob. Agents Chemother.* 20 244–246. 10.1128/aac.20.2.244 7025754 PMC181671

[B95] Ito A. Nishikawa T. Matsumoto S. Yoshizawa H. Sato T. Nakamura R. (2016). Siderophore cephalosporin cefiderocol utilizes ferric iron transporter systems for antibacterial activity against *Pseudomonas aeruginosa*. *Antimicrob. Agents Chemother.* 60 7396–7401. 10.1128/AAC.01405-16 27736756 PMC5119021

[B96] James M. N. Hall D. Hodgkin D. C. (1968). Crystalline modifications of ampicillin I: the trihydrate. *Nature* 220 168–170. 10.1038/220168a0 5684826

[B97] Jean S. S. Lee W. S. Ko W. C. Hsueh P. R. (2020). *In vitro* susceptibility of ceftaroline against clinically important Gram-positive cocci, *Haemophilus* species and *Klebsiella pneumoniae* in Taiwan: results from the antimicrobial testing leadership and surveillance (ATLAS) in 2012-2018. *J. Microbiol. Immunol. Infect.* 54 627–631. 10.1016/j.jmii.2020.04.017 32451293

[B98] Jeong J. H. Cha H. J. Kim Y. G. (2018). Crystal structures of penicillin-binding protein D2 from *Listeria monocytogenes* and structural basis for antibiotic specificity. *Antimicrob. Agents Chemother.* 62:e00796-18. 10.1128/AAC.00796-18 30082290 PMC6125574

[B99] Jones R. N. Barry A. L. Thornsberry C. Wilson H. W. (1981). In vitro antimicrobial activity evaluation of cefodizime (HR221), a new semisynthetic cephalosporin. *Antimicrob. Agents Chemother.* 20 760–768. 10.1128/aac.20.6.760 6275785 PMC181795

[B100] Jones R. N. Thornsberry C. Barry A. L. (1984). In vitro evaluation of HR810, a new wide-spectrum aminothiazolyl alpha-methoxyimino cephalosporin. *Antimicrob. Agents Chemother.* 25 710–718. 10.1128/aac.25.6.710 6611135 PMC185628

[B101] Kamimura T. Kojo H. Matsumoto Y. Mine Y. Goto S. Kuwahara S. (1984). In vitro and in vivo antibacterial properties of FK 027, a new orally active cephem antibiotic. *Antimicrob. Agents Chemother.* 25 98–104. 10.1128/aac.25.1.98 6561017 PMC185443

[B102] Kamimura T. Matsumoto Y. Okada N. Mine Y. Nishida M. Goto S. (1979). Ceftizoxime (FK 749), a new parenteral cephalosporin: in vitro and in vivo antibacterial activities. *Antimicrob. Agents Chemother.* 16 540–548. 10.1128/aac.16.5.540 525994 PMC352902

[B103] Kayser F. H. Morenzoni G. Strassle A. Hadorn K. (1989). Activity of meropenem, against gram-positive bacteria. *J. Antimicrob. Chemother.* 24 (Suppl. A), 101–112. 10.1093/jac/24.suppl_a.101 2808202

[B104] Kessler R. E. Bies M. Buck R. E. Chisholm D. R. Pursiano T. A. Tsai Y. H. (1985). Comparison of a new cephalosporin, BMY 28142, with other broad-spectrum beta-lactam antibiotics. *Antimicrob. Agents Chemother.* 27 207–216. 10.1128/aac.27.2.207 3885849 PMC176239

[B105] Khan N. J. Bihl J. A. Schell R. F. LeFrock J. L. Weber S. J. (1984). Antimicrobial activities of BMY-28142, cefbuperazone, and cefpiramide compared with those of other cephalosporins. *Antimicrob. Agents Chemother.* 26 585–590. 10.1128/aac.26.4.585 6549120 PMC179970

[B106] Kivits T. Broers H. P. Beeltje H. van Vliet M. Griffioen J. (2018). Presence and fate of veterinary antibiotics in age-dated groundwater in areas with intensive livestock farming. *Environ. Pollut.* 241 988–998. 10.1016/j.envpol.2018.05.085 30029333

[B107] Knox R. (1961). A survey of new penicillins. *Nature* 192 492–496. 10.1038/192492a0 14457375

[B108] Kohira N. West J. Ito A. Ito-Horiyama T. Nakamura R. Sato T. (2016). In vitro antimicrobial activity of a siderophore cephalosporin, S-649266, against *Enterobacteriaceae* clinical isolates, including carbapenem-resistant strains. *Antimicrob. Agents Chemother.* 60 729–734. 10.1128/AAC.01695-15 26574013 PMC4750680

[B109] Kojo H. Nishida M. Goto S. Kuwahara S. (1979). Antibacterial activity of ceftizoxime (FK 749), a new cephalosporin, against cephalosporin-resistant bacteria, and its stability to beta-lactamase. *Antimicrob. Agents Chemother.* 16 549–553. 10.1128/aac.16.5.549 316684 PMC352903

[B110] Kosmidis J. Hamilton-Miller J. M. Gilchrist J. N. Kerry D. W. Brumfitt W. (1973). Cefoxitin, a new semi-synthetic cephamycin: an in-vitro and in-vivo comparison with cephalothin. *Br. Med. J.* 4 653–655. 10.1136/bmj.4.5893.653 4202265 PMC1587624

[B111] Kriebernegg I. Feierl G. Grisold A. Marth E. (1998). In-vitro susceptibility of group A beta-haemolytic streptococci (GABHS) to penicillin, erythromycin, clarithromycin and azithromycin in Styria, Austria. *Zentralbl. Bakteriol.* 287 33–39. 10.1016/s0934-8840(98)80139-4 9532262

[B112] Lang M. Prasad K. Holick W. Gosteli J. Ernest I. Woodward R. B. (1978). The penems, a new class of beta-lactam antibiotics. 2. Total synthesis of racemic 6-unsubstituted representatives. *J. Am. Chem. Soc.* 101 6296–6301.

[B113] Lev E. I. Kleiman N. S. Birnbaum Y. Harris D. Korbling M. Estrov Z. (2005). Circulating endothelial progenitor cells and coronary collaterals in patients with non-ST segment elevation myocardial infarction. *J. Vasc. Res.* 42 408–414. 10.1159/000087370 16088214

[B114] Libby R. L. Holmberg N. L. (1945). The activity of penicillins G and X *in vitro*. *Science* 102 303–304. 10.1126/science.102.2647.303 17829678

[B115] Lima L. M. Silva B. Barbosa G. Barreiro E. J. (2020). beta-lactam antibiotics: an overview from a medicinal chemistry perspective. *Eur. J. Med. Chem.* 208:112829. 10.1016/j.ejmech.2020.112829 33002736

[B116] Limbert M. Isert D. Klesel N. Markus A. Seeger K. Seibert G. (1991). Antibacterial activities in vitro and in vivo and pharmacokinetics of cefquinome (HR 111V), a new broad-spectrum cephalosporin. *Antimicrob. Agents Chemother.* 35 14–19. 10.1128/aac.35.1.14 2014969 PMC244934

[B117] Livermore D. M. Carter M. W. Bagel S. Wiedemann B. Baquero F. Loza E. (2001). In vitro activities of ertapenem (MK-0826) against recent clinical bacteria collected in Europe and Australia. *Antimicrob. Agents Chemother.* 45 1860–1867. 10.1128/AAC.45.6.1860-1867.2001 11353638 PMC90558

[B118] Lohans C. T. Chan H. T. H. Malla T. R. Kumar K. Kamps J. McArdle D. J. B. (2019). Non-hydrolytic beta-lactam antibiotic fragmentation by l,d-transpeptidases and serine beta-lactamase cysteine variants. *Angew. Chem. Int. Ed Engl.* 58 1990–1994. 10.1002/anie.201809424 30569575 PMC6391942

[B119] Lorcheim K. (2011). Chlorine dioxide gas inactivation of beta-lactams. *Appl. Biosaf.* 16 34–43.

[B120] Lu Z. Wang H. Zhang A. Liu X. Zhou W. Yang C. (2020). Structures of *Mycobacterium tuberculosis* penicillin-binding protein 3 in complex with five beta-lactam antibiotics reveal mechanism of inactivation. *Mol. Pharmacol.* 97 287–294. 10.1124/mol.119.118042 32086254

[B121] Macheboeuf P. Contreras-Martel C. Job V. Dideberg O. Dessen A. (2006). Penicillin binding proteins: key players in bacterial cell cycle and drug resistance processes. *FEMS Microbiol. Rev.* 30 673–691. 10.1111/j.1574-6976.2006.00024.x 16911039

[B122] Machka K. Braveny I. (1983). In vitro activity of HR 810, a new broad-spectrum cephalosporin. *Eur. J. Clin. Microbiol.* 2 345–349. 10.1007/BF02019465 6313358

[B123] Macris M. H. Hartman N. Murray B. Klein R. F. Roberts R. B. Kaplan E. L. (1998). Studies of the continuing susceptibility of group A streptococcal strains to penicillin during eight decades. *Pediatr. Infect. Dis. J.* 17 377–381. 10.1097/00006454-199805000-00006 9613649

[B124] Marchese A. Gualco L. Schito A. M. Debbia E. A. Schito G. C. (2004). In vitro activity of ertapenem against selected respiratory pathogens. *J. Antimicrob. Chemother.* 54 944–951. 10.1093/jac/dkh445 15472001

[B125] Matsubara N. Minami S. Muraoka T. Saikawa I. Mitsuhashi S. (1979). In vitro antibacterial activity of cefoperazone (T-1551), a new semisynthetic cephalosporin. *Antimicrob. Agents Chemother.* 16 731–735. 10.1128/aac.16.6.731 316988 PMC352944

[B126] Meng G. Zhang J. Szostak M. (2021). Acyclic twisted amides. *Chem. Rev.* 121 12746–12783. 10.1021/acs.chemrev.1c00225 34406005 PMC9108997

[B127] Mine Y. Kamimura T. Watanabe Y. Tawara S. Matsumoto Y. Shibayama F. (1988). In vitro antibacterial activity of FK482, a new orally active cephalosporin. *J. Antibiot.* 41 1873–1887. 10.7164/antibiotics.41.1873 3264828

[B128] Morin R. B. Jackson B. G. Mueller R. A. Lavagnino E. R. Scanlon W. B. Andrews S. L. (1969). Chemistry of cephalosporin antibiotics. XV. Transformations of penicillin sulfoxide. A synthesis of cephalosporin compounds. *J. Am. Chem. Soc.* 91 1401–1407. 10.1021/ja01034a023 5776255

[B129] Musser J. M. Beres S. B. Zhu L. Olsen R. J. Vuopio J. Hyyrylainen H. L. (2020). Reduced *in vitro* susceptibility of *Streptococcus pyogenes* to beta-lactam antibiotics associated with mutations in the pbp2x gene is geographically widespread. *J. Clin. Microbiol.* 58:e01993-19. 10.1128/JCM.01993-19 31996443 PMC7098749

[B130] Nell E. E. Hill J. H. (1947). Comparison of the in vitro antigonococcal actions of penicillins G, F, K, and X. *Am. J. Syph. Gonorrhea Vener. Dis.* 31 14–19. 20281480

[B131] Neu H. C. (1974b). Cefoxitin, a semisynthetic cephamycin antibiotic: antibacterial spectrum and resistance to hydrolysis by gram-negative beta-lactamases. *Antimicrob. Agents Chemother.* 6 170–176. 10.1128/aac.6.2.170 15828188 PMC444623

[B132] Neu H. C. (1974a). Cefamandole, a cephalosporin antibiotic with an unusually wide spectrum of activity. *Antimicrob. Agents Chemother.* 6 177–182. 10.1128/aac.6.2.177 15828189 PMC444624

[B133] Neu H. C. (1976). Mecillinam, a novel penicillanic acid derivative with unusual activity against gram-negative bacteria. *Antimicrob. Agents Chemother.* 9 793–799. 10.1128/aac.9.5.793 949176 PMC429623

[B134] Neu H. C. Chin N. X. (1986). In vitro activity and beta-lactamase stability of a new difluoro oxacephem, 6315-S. *Antimicrob. Agents Chemother.* 30 638–644. 10.1128/aac.30.5.638 3492172 PMC176505

[B135] Neu H. C. Chin N. X. Labthavikul P. (1984). Comparative in vitro activity and beta-lactamase stability of FR 17027, a new orally active cephalosporin. *Antimicrob. Agents Chemother.* 26 174–180. 10.1128/aac.26.2.174 6333207 PMC284114

[B136] Neu H. C. Fu K. P. Aswapokee N. Aswapokee P. Kung K. (1979). Comparative activity and beta-lactamase stability of cefoperazone, a piperazine cephalosporin. *Antimicrob. Agents Chemother.* 16 150–157. 10.1128/aac.16.2.150 314775 PMC352812

[B137] Neu H. C. Meropol N. J. Fu K. P. (1981). Antibacterial activity of ceftriaxone (Ro 13-9904), a beta-lactamase-stable cephalosporin. *Antimicrob. Agents Chemother.* 19 414–423. 10.1128/aac.19.3.414 6972729 PMC181447

[B138] Neu H. C. Saha G. Chin N. X. (1989). Comparative in vitro activity and beta-lactamase stability of FK482, a new oral cephalosporin. *Antimicrob. Agents Chemother.* 33 1795–1800. 10.1128/aac.33.10.1795 2589845 PMC172757

[B139] Nomura H. Fugono T. Hitaka T. Minami I. Azuma T. Morimoto S. (1974). Semisynthetic beta-lactam antibiotics. 6. 1 Sulfocephalosporins and their antipseudomonal activities. *J. Med. Chem.* 17 1312–1315. 10.1021/jm00258a017 4214929

[B140] O’Callaghan C. H. Acred P. Harper P. B. Ryan D. M. Kirby S. M. Harding S. M. (1980). GR 20263, a new broad-spectrum cephalosporin with anti-pseudomonal activity. *Antimicrob. Agents Chemother.* 17 876–883. 10.1128/aac.17.5.876 6994642 PMC283891

[B141] Ochoa-Aguilar A. Ventura-Martinez R. Sotomayor-Sobrino M. A. Gomez C. Morales-Espinoza M. R. (2016). Review of antibiotic and non-antibiotic properties of beta-lactam molecules. *Antiinflamm. Antiallergy Agents Med. Chem.* 15 3–14. 10.2174/1871523015666160517114027 27185396

[B142] Okamoto S. Hamana Y. Inoue M. Mitsuhashi S. (1987). In vitro and in vivo antibacterial activities of T-2588, a new oral cephalosporin, compared with those of other oral beta-lactam antibiotics. *Antimicrob. Agents Chemother.* 31 1111–1116. 10.1128/aac.31.7.1111 3499115 PMC174880

[B143] Osborn M. Stachulski N. Sun H. Blais J. Venishetty V. Raccuglia M. (2019). A first-in-human study to assess the safety and pharmacokinetics of LYS228, a novel intravenous monobactam antibiotic in healthy volunteers. *Antimicrob. Agents Chemother.* 63:e02592-18. 10.1128/AAC.02592-18 31061156 PMC6591616

[B144] Pechere J. C. Kohler T. (1999). Patterns and modes of beta-lactam resistance in *Pseudomonas aeruginosa*. *Clin. Microbiol. Infect.* 5 (Suppl. 1), S15–S18. 10.1111/j.1469-0691.1999.tb00719.x 11869272

[B145] Pendland S. L. Neuhauser M. M. Prause J. L. (2002). In vitro bactericidal activity of ABT-773 and amoxicillin against erythromycin-susceptible and -resistant strains of *Streptococcus pyogenes*. *J. Antimicrob. Chemother.* 49 671–674. 10.1093/jac/49.4.671 11909842

[B146] Peterson Y. K. Cameron R. B. Wills L. P. Trager R. E. Lindsey C. C. Beeson C. C. (2013). beta-adrenoceptor agonists in the regulation of mitochondrial biogenesis. *Bioorg. Med. Chem. Lett.* 23 5376–5381. 10.1016/j.bmcl.2013.07.052 23954364 PMC3987705

[B147] Poch G. Brunner F. Kuhberger E. (1992). Construction of antagonist dose-response curves for estimation of pA2-values by schild-plot analysis and detection of allosteric interactions. *Br. J. Pharmacol.* 106 710–716. 10.1111/j.1476-5381.1992.tb14399.x 1504755 PMC1907557

[B148] Reiner R. Weiss U. Brombacher U. Lanz P. Montavon M. Furlenmeier A. (1980). Ro 13-9904/001, a novel potent and long-acting parenteral cephalosporin. *J. Antibiot.* 33 783–786. 10.7164/antibiotics.33.783 6967869

[B149] Rittenbury M. S. (1990). How and why aztreonam works. *Surg. Gynecol. Obstet.* 171 (Suppl.), 19–23. 2244291

[B150] Romano A. Gaeta F. Valluzzi R. L. Zaffiro A. Caruso C. Quaratino D. (2014). Natural evolution of skin-test sensitivity in patients with IgE-mediated hypersensitivity to cephalosporins. *Allergy* 69 806–809. 10.1111/all.12390 24673580

[B151] Romano A. Gueant-Rodriguez R. M. Viola M. Pettinato R. Gueant J. L. (2004). Cross-reactivity and tolerability of cephalosporins in patients with immediate hypersensitivity to penicillins. *Ann. Intern. Med.* 141 16–22. 10.7326/0003-4819-141-1-200407060-00010 15238366

[B152] Romano A. Valluzzi R. L. Caruso C. Maggioletti M. Quaratino D. Gaeta F. (2018). Cross-reactivity and tolerability of cephalosporins in patients with IgE-mediated hypersensitivity to penicillins. *J. Allergy Clin. Immunol. Pract.* 6 1662–1672. 10.1016/j.jaip.2018.01.020 29408440

[B153] Romano A. Viola M. Gueant-Rodriguez R. M. Gaeta F. Pettinato R. Gueant J. L. (2006). Imipenem in patients with immediate hypersensitivity to penicillins. *N. Engl. J. Med.* 354 2835–2837. 10.1056/NEJMc053529 16807429

[B154] Romano A. Viola M. Gueant-Rodriguez R. M. Gaeta F. Valluzzi R. Gueant J. L. (2007). Brief communication: tolerability of meropenem in patients with IgE-mediated hypersensitivity to penicillins. *Ann. Intern. Med.* 146 266–269. 10.7326/0003-4819-146-4-200702200-00005 17310050

[B155] Ryan C. W. Simon R. L. Van Heyningen E. M. (1969). Chemistry of cephalosporin antibiotics. 13. Desacetoxycephalosporins. The synthesis of cephalexin and some analogs. *J. Med. Chem.* 12 310–313. 10.1021/jm00302a026 5783607

[B156] Sader H. S. Fritsche T. R. Kaniga K. Ge Y. Jones R. N. (2005). Antimicrobial activity and spectrum of PPI-0903M (T-91825), a novel cephalosporin, tested against a worldwide collection of clinical strains. *Antimicrob. Agents Chemother.* 49 3501–3512. 10.1128/AAC.49.8.3501-3512.2005 16048970 PMC1196228

[B157] Sakagawa E. Otsuki M. Oh T. Nishino T. (1998). In-vitro and in-vivo antibacterial activities of CS-834, a new oral carbapenem. *J. Antimicrob. Chemother.* 42 427–437. 10.1093/jac/42.4.427 9818740

[B158] Sakata H. (2013). Susceptibility and emm type of *Streptococcus pyogenes* isolated from children with severe infection. *J. Infect. Chemother.* 19 1042–1046. 10.1007/s10156-013-0617-6 23703641 PMC3855535

[B159] Sato J. Kusano H. Aoki T. Shibuya S. Yokoo K. Komano K. (2022). Discovery of a tricyclic beta-lactam as a potent antimicrobial agent against carbapenem-resistant Enterobacterales, including strains with reduced membrane permeability and four-amino acid insertion into penicillin-binding protein 3: structure-activity-relationships and *in vitro* and *in vivo* activities. *ACS Infect. Dis.* 10.1021/acsinfecdis.1c00549 [Online ahead of print]. 35112852

[B160] Sauvage E. Kerff F. Terrak M. Ayala J. A. Charlier P. (2008). The penicillin-binding proteins: structure and role in peptidoglycan biosynthesis. *FEMS Microbiol. Rev.* 32 234–258. 10.1111/j.1574-6976.2008.00105.x 18266856

[B161] Scully B. E. Jules K. Neu H. C. (1983). In vitro activity and beta-lactamase stability of cefodizime, an aminothiazolyl iminomethoxy cephalosporin. *Antimicrob. Agents Chemother.* 23 907–913. 10.1128/aac.23.6.907 6311090 PMC185001

[B162] Seibert G. Klesel N. Limbert M. Schrinner E. Seeger K. Winkler I. (1983). HR 810, a new parenteral cephalosporin with a broad antibacterial spectrum. *Arzneimittelforschung* 33 1084–1086. 10.1002/chin.198350201 6315023

[B163] Shannon K. King A. Warren C. Phillips I. (1980). In vitro antibacterial activity and susceptibility of the cephalosporin Ro 13-9904 to beta-lactamases. *Antimicrob. Agents Chemother.* 18 292–298. 10.1128/aac.18.2.292 6969574 PMC283986

[B164] Shapiro A. B. Moussa S. H. McLeod S. M. Durand-Reville T. Miller A. A. (2021). Durlobactam, a new diazabicyclooctane beta-lactamase inhibitor for the treatment of acinetobacter infections in combination with sulbactam. *Front. Microbiol.* 12:709974. 10.3389/fmicb.2021.709974 34349751 PMC8328114

[B165] Shelton S. Nelson J. D. (1988). In vitro susceptibilities of common pediatric pathogens to LY163892. *Antimicrob. Agents Chemother.* 32 268–270. 10.1128/aac.32.2.268 3129989 PMC172150

[B166] Shibata M. Kashiwagi Y. Tokue S. Tominaga Y. Okubo M. (1975). [Drug susceptibility of hemolytic streptococci isolated from patients with scarlet fever during 1973]. *Kansenshogaku Zasshi* 49 94–97. 10.11150/kansenshogakuzasshi1970.49.94 811726

[B167] Sidell S. Burdick R. E. Brodie J. Bulger R. J. Kirby W. M. (1963). New antistaphylococcal antibiotics. Comparative in vitro and in vivo activity of cephalothin, nafcillin, cloxacillin, oxacillin, and methicillin. *Arch. Intern. Med.* 112 21–28. 10.1001/archinte.1963.03860010067005 13988897

[B168] Silvaggi N. R. Josephine H. R. Kuzin A. P. Nagarajan R. Pratt R. F. Kelly J. A. (2005). Crystal structures of complexes between the R61 DD-peptidase and peptidoglycan-mimetic beta-lactams: a non-covalent complex with a “perfect penicillin”. *J. Mol. Biol.* 345 521–533. 10.1016/j.jmb.2004.10.076 15581896

[B169] Singh J. Arrieta A. C. (1999). New cephalosporins. *Semin. Pediatr. Infect. Dis.* 10 14–22.

[B170] Song Z. Zhang X. Ngo H. H. Guo W. Wen H. Li C. (2019). Occurrence, fate and health risk assessment of 10 common antibiotics in two drinking water plants with different treatment processes. *Sci. Total Environ.* 674 316–326. 10.1016/j.scitotenv.2019.04.093 31005833

[B171] Sosna J. P. Murray P. R. Medoff G. (1978). Comparison of the in vitro activities of HR756 with cephalothin, cefoxitin, and cefamandole. *Antimicrob. Agents Chemother.* 14 876–879. 10.1128/aac.14.6.876 742875 PMC352572

[B172] Spencer J. L. Flynn E. H. Roeske R. W. Siu F. Y. Chauvette R. R. (1966a). Chemistry of cephalosporin antibiotics. VII. Synthesis of cephaloglycin and some homologs. *J. Med. Chem.* 9 746–750. 10.1021/jm00323a024 5182352

[B173] Spencer J. L. Siu F. Y. Flynn E. H. Jackson B. G. Sigal M. V. Higgins H. M. (1966b). Chemistry of cephalosporin antibiotics. 8. Synthesis and structure-activity relationships of cephaloridine analogues. *Antimicrob. Agents Chemother.* 6 573–580. 5985291

[B174] Stamm J. M. Girolami R. L. Shipkowitz N. L. Bower R. R. (1981). Antimicrobial activity of cefmenoxime (SCE-1365). *Antimicrob. Agents Chemother.* 19 454–460. 10.1128/aac.19.3.454 6264846 PMC181453

[B175] Stefani S. Mezzatesta M. L. Fadda G. Mattina R. Palu G. Rossano F. (2008). Antibacterial activity of cefditoren against major community-acquired respiratory pathogens recently isolated in Italy. *J. Chemother.* 20 561–569. 10.1179/joc.2008.20.5.561 19028617

[B176] Stewart D. Bodey G. P. (1977). Azlocillin: in vitro studies of a new semisynthetic penicillin. *Antimicrob. Agents Chemother.* 11 865–870. 10.1128/aac.11.5.865 18083 PMC352088

[B177] Stover K. R. Barber K. E. Wagner J. L. (2019). Allergic reactions and cross-reactivity potential with beta-lactamase inhibitors. *Pharmacy* 7:77. 10.3390/pharmacy7030077 31261671 PMC6789713

[B178] Suzuki Y. Ishihara R. Ishii Y. Nakazawa A. Deguchi K. Matsumoto Y. (1996). [Antimicrobial activity of cefodizime against clinical isolates]. *Jpn. J. Antibiot.* 49 947–965. 8986558

[B179] Sweet R. M. Dahl L. F. (1970). Molecular architecture of the cephalosporins. Insights into biological activity based on structural investigations. *J. Am. Chem. Soc.* 92 5489–5507. 10.1021/ja00721a032 5449447

[B180] Sykes R. B. Bonner D. P. Bush K. Georgopapadakou N. H. (1982). Azthreonam (SQ 26,776), a synthetic monobactam specifically active against aerobic gram-negative bacteria. *Antimicrob. Agents Chemother.* 21 85–92. 10.1128/aac.21.1.85 6979307 PMC181833

[B181] Sykes R. B. Cimarusti C. M. Bonner D. P. Bush K. Floyd D. M. Georgopapadakou N. H. (1981). Monocyclic beta-lactam antibiotics produced by bacteria. *Nature* 291 489–491. 10.1038/291489a0 7015152

[B182] Szekeres E. Chiriac C. M. Baricz A. Szoke-Nagy T. Lung I. Soran M. L. (2018). Investigating antibiotics, antibiotic resistance genes, and microbial contaminants in groundwater in relation to the proximity of urban areas. *Environ. Pollut.* 236 734–744. 10.1016/j.envpol.2018.01.107 29454283

[B183] Szostak R. Aube J. Szostak M. (2015). Determination of structures and energetics of small- and medium-sized one-carbon-bridged twisted amides using AB initio molecular orbital methods: implications for amidic resonance along the C-N rotational pathway. *J. Org. Chem.* 80 7905–7927. 10.1021/acs.joc.5b00881 26154179

[B184] Takeda S. Ishii Y. Hatano K. Tateda K. Yamaguchi K. (2007a). Stability of FR264205 against AmpC beta-lactamase of *Pseudomonas aeruginosa*. *Int. J. Antimicrob. Agents* 30 443–445. 10.1016/j.ijantimicag.2007.05.019 17644319

[B185] Takeda S. Nakai T. Wakai Y. Ikeda F. Hatano K. (2007b). In vitro and in vivo activities of a new cephalosporin, FR264205, against *Pseudomonas aeruginosa*. *Antimicrob. Agents Chemother.* 51 826–830. 10.1128/AAC.00860-06 17145788 PMC1803152

[B186] Tao W. Ivanovska V. Schweickert B. Muller A. (2019). Proxy indicators for antibiotic consumption; surveillance needed to control antimicrobial resistance. *Bull. World Health Organ.* 97 3–3A. 10.2471/BLT.18.227348 30618457 PMC6307504

[B187] Tempera G. Furneri P. M. Carlone N. A. Cocuzza C. Rigoli R. Musumeci R. (2010). Antibiotic susceptibility of respiratory pathogens recently isolated in Italy: focus on cefditoren. *J. Chemother.* 22 153–159. 10.1179/joc.2010.22.3.153 20566418

[B188] Thomsen V. F. Larsen S. O. (1962). [In vitro activity of penicillin. Comparative studies on benzylpenicillin, phenoxymethylpenicillin, phenoxyethylpenicillin and dimethoxyphenylpenicillin]. *Nord. Med.* 67 111–119. 13920960

[B189] Thornhill T. S. Levison M. E. Johnson W. D. Kaye D. (1969). In vitro antimicrobial activity and human pharmacology of cephalexin, a new orally absorbed cephalosporin C antibiotic. *Appl. Microbiol.* 17 457–461. 4388601 10.1128/am.17.3.457-461.1969 PMC377712

[B190] Toda A. Ohki H. Yamanaka T. Murano K. Okuda S. Kawabata K. (2008). Synthesis and SAR of novel parenteral anti-pseudomonal cephalosporins: discovery of FR264205. *Bioorg. Med. Chem. Lett.* 18 4849–4852. 10.1016/j.bmcl.2008.07.085 18701284

[B191] Toda M. Arao N. Nohara C. Susaki K. Tachibana A. (1985). In vitro studies on the antibacterial activities of YM-13115, a new broad-spectrum cephalosporin. *Antimicrob. Agents Chemother.* 27 565–569. 10.1128/aac.27.4.565 3890729 PMC180096

[B192] Trubiano J. A. Stone C. A. Grayson M. L. Urbancic K. Slavin M. A. Thursky K. A. (2017). The 3 Cs of antibiotic allergy-classification, cross-reactivity, and collaboration. *J. Allergy Clin. Immunol. Pract.* 5 1532–1542. 10.1016/j.jaip.2017.06.017 28843343 PMC5681410

[B193] Tsuchiya K. Kondo M. Kida M. Nakao M. Iwahi T. Nishi T. (1981). Cefmenoxime (SCE-1365), a novel broad-spectrum cephalosporin: in vitro and in vivo antibacterial activities. *Antimicrob. Agents Chemother.* 19 56–65. 10.1128/aac.19.1.56 6941742 PMC181357

[B194] Tsuchiya K. Kondo M. Nagatomo H. (1978). SCE-129, antipseudomonal cephalosporin: in vitro and in vivo antibacterial activities. *Antimicrob. Agents Chemother.* 13 137–145. 10.1128/aac.13.2.137 417670 PMC352203

[B195] Tsuji M. Ishii Y. Ohno A. Miyazaki S. Yamaguchi K. (1995). In vitro and in vivo antibacterial activities of S-1090, a new oral cephalosporin. *Antimicrob. Agents Chemother.* 39 2544–2551. 10.1128/aac.39.11.2544 8585742 PMC162981

[B196] Tsuji M. Ishii Y. Ohno A. Miyazaki S. Yamaguchi K. (1998). In vitro and in vivo antibacterial activities of S-4661, a new carbapenem. *Antimicrob. Agents Chemother.* 42 94–99. 10.1128/AAC.42.1.94 9449267 PMC105462

[B197] Ubukata K. Wajima T. Morozumi M. Sakuma M. Tajima T. Matsubara K. (2020). Changes in epidemiologic characteristics and antimicrobial resistance of *Streptococcus pyogenes* isolated over 10 years from Japanese children with pharyngotonsillitis. *J. Med. Microbiol.* 69 443–450. 10.1099/jmm.0.001158 32011228

[B198] Une T. Otani T. Sato M. Ikeuchi T. Osada Y. Ogawa H. (1985). In vitro and in vivo activities of DN-9550, a new broad-spectrum cephalosporin. *Antimicrob. Agents Chemother.* 27 473–478. 10.1128/aac.27.4.473 3873898 PMC180077

[B199] Veeraraghavan B. Bakthavatchalam Y. D. Sahni R. D. (2021). Orally administered amoxicillin/clavulanate: current role in outpatient therapy. *Infect. Dis. Ther.* 10 15–25. 10.1007/s40121-020-00374-7 33306184 PMC7954971

[B200] Verbist L. (1978). In vitro activity of piperacillin, a new semisynthetic penicillin with an unusually broad spectrum of activity. *Antimicrob. Agents Chemother.* 13 349–357. 10.1128/aac.13.3.349 122518 PMC352245

[B201] Verbist L. Verhaegen J. (1980). GR-20263: a new aminothiazolyl cephalosporin with high activity against *Pseudomonas* and *Enterobacteriaceae*. *Antimicrob. Agents Chemother.* 17 807–812. 10.1128/aac.17.5.807 6994640 PMC283880

[B202] Vuye A. Pijck J. (1985). In vitro antibacterial activity of BMY-28142, a new extended-spectrum cephalosporin. *Antimicrob. Agents Chemother.* 27 574–577. 10.1128/aac.27.4.574 3859244 PMC180098

[B203] Wallick H. Hendlin D. (1974). Cefoxitin, a semisynthetic cephamycin antibiotic: susceptibility studies. *Antimicrob. Agents Chemother.* 5 25–32. 10.1128/aac.5.1.25 4840447 PMC428914

[B204] Wallmark G. (1962). Comparison in vitro of new penicillins. Special reference to methicillin-resistant staphylococci. *Arch. Intern. Med.* 110 787–800. 10.1001/archinte.1962.03620230233032 13998620

[B205] Watanakunakorn C. Glotzbecker C. (1979). Comparative in vitro activity of LY 127935 (6059-S), seven cephalosporins, three aminoglycosides, carbenicillin, and ticarcillin. *J. Antibiot.* 32 1019–1024. 10.7164/antibiotics.32.1019 528361

[B206] Waxman D. J. Yocum R. R. Strominger J. L. (1980). Penicillins and cephalosporins are active site-directed acylating agents: evidence in support of the substrate analogue hypothesis. *Philos. Trans. R. Soc. Lond. B Biol. Sci.* 289 257–271. 10.1098/rstb.1980.0044 6109322

[B207] Weaver S. S. Bodey G. P. LeBlanc B. M. (1979). Thienamycin: new beta-lactam antibiotic with potent broad-spectrum activity. *Antimicrob. Agents Chemother.* 15 518–521. 10.1128/aac.15.4.518 380462 PMC352703

[B208] Wise R. Andrews J. M. Ashby J. P. Matthews R. S. (1988). In vitro activity of lomefloxacin, a new quinolone antimicrobial agent, in comparison with those of other agents. *Antimicrob. Agents Chemother.* 32 617–622. 10.1128/aac.32.5.617 3134843 PMC172240

[B209] Wise R. Andrews J. M. Ashby J. P. Thornber D. (1991). Ceftibuten: a new orally absorbed cephalosporin. In vitro activity against strains from the United Kingdom. *Diagn. Microbiol. Infect. Dis.* 14 45–52. 10.1016/0732-8893(91)90089-x 2013210

[B210] Wise R. Andrews J. M. Bedford K. A. (1980). Comparison of in vitro activity of GR 20263, a novel cephalosporin derivative, with activities of other beta-lactam compounds. *Antimicrob. Agents Chemother.* 17 884–889. 10.1128/aac.17.5.884 6772097 PMC283892

[B211] Wise R. Rollason T. Logan M. Andrews J. M. Bedford K. A. (1978). HR 756, a highly active cephalosporin: comparison with cefazolin and carbenicillin. *Antimicrob. Agents Chemother.* 14 807–811. 10.1128/aac.14.6.807 253572 PMC352561

[B212] Woodcock J. M. Andrews J. M. Brenwald N. P. Ashby J. P. Wise R. (1997). The in-vitro activity of faropenem, a novel oral penem. *J. Antimicrob. Chemother.* 39 35–43. 10.1093/jac/39.1.35 9044026

[B213] Woodward R. B. (1980). Penems and related substances. *Philos. Trans. R. Soc. Lond. B Biol. Sci.* 289 239–250. 10.1098/rstb.1980.0042 6109320

[B214] Wootton M. Bowker K. E. Holt H. A. MacGowan A. P. (2002). BAL 9141, a new broad-spectrum pyrrolidinone cephalosporin: activity against clinically significant anaerobes in comparison with 10 other antimicrobials. *J. Antimicrob. Chemother.* 49 535–539. 10.1093/jac/49.3.535 11864955

[B215] Yamaguchi K. Domon H. Miyazaki S. Tateda K. Ohno A. Ishii K. (1998). In vitro and in vivo antibacterial activities of CS-834, a new oral carbapenem. *Antimicrob. Agents Chemother.* 42 555–563. 10.1128/AAC.42.3.555 9517932 PMC105498

[B216] Yan S. Mendelman P. M. Stevens D. L. (1993). The in vitro antibacterial activity of ceftriaxone against *Streptococcus pyogenes* is unrelated to penicillin-binding protein 4. *FEMS Microbiol. Lett.* 110 313–317. 10.1111/j.1574-6968.1993.tb06341.x 8354465

[B217] Yanagihara K. Matsumoto T. Tokimatsu I. Tsukada H. Fujikura Y. Miki M. (2020). Nationwide surveillance of bacterial respiratory pathogens conducted by the surveillance committee of Japanese society of chemotherapy, the Japanese association for infectious diseases, and the Japanese society for clinical microbiology in 2016: general view of the pathogens’ antibacterial susceptibility. *J. Infect. Chemother.* 26 873–881. 10.1016/j.jiac.2020.05.006 32565151

[B218] Yang Q. Zhang H. Cheng J. Xu Z. Hou X. Xu Y. (2015). Flomoxef showed excellent in vitro activity against clinically important gram-positive and gram-negative pathogens causing community- and hospital-associated infections. *Diagn. Microbiol. Infect. Dis.* 81 269–274. 10.1016/j.diagmicrobio.2015.01.001 25641126

[B219] Yanik K. Guluzade E. Bilgin K. Karadag A. Eroglu C. Birinci A. (2015). Ceftaroline activity on certain respiratory tract and wound infection agents at the minimum inhibitory concentration level. *J. Infect. Dev. Ctries.* 9 1086–1090. 10.3855/jidc.6300 26517483

[B220] Yazawa K. Mikami Y. Uno J. Otozai K. Arai T. (1989). In-vitro activity of flomoxef, a new oxacephem group antibiotic, against Nocardia in comparison with other cephalosporins. *J. Antimicrob. Chemother.* 24 921–925. 10.1093/jac/24.6.921 2621177

[B221] Yocum R. R. Waxman D. J. Rasmussen J. R. Strominger J. L. (1979). Mechanism of penicillin action: penicillin and substrate bind covalently to the same active site serine in two bacterial D-alanine carboxypeptidases. *Proc. Natl. Acad. Sci. U.S.A.* 76 2730–2734. 10.1073/pnas.76.6.2730 111240 PMC383682

[B222] Yokoo C. Goi M. Onodera A. Fukushima H. Nagate T. (1991). Studies on cephalosporin antibiotics. IV. Synthesis, antibacterial activity and oral absorption of new 3-(2-substituted-vinylthio)-7 beta-[(Z)-2-(2-aminothiazol-4-yl)-2- (carboxymethoxyimino)acetamido]cephalosporins. *J. Antibiot.* 44 1422–1431. 10.7164/antibiotics.44.1422 1778793

[B223] Yu K. W. Neu H. C. (1989). Antibacterial activity and beta-lactamase stability of MDL 19,592, an oral cephalosporin. *Diagn. Microbiol. Infect. Dis.* 12 441–443. 10.1016/0732-8893(89)90117-x 2515027

[B224] Zagursky R. J. Pichichero M. E. (2018). Cross-reactivity in beta-lactam allergy. *J. Allergy Clin. Immunol. Pract.* 6 72–81.e1. 10.1016/j.jaip.2017.08.027 29017833

[B225] Zbinden R. Punter V. von Graevenitz A. (2002). In vitro activities of BAL9141, a novel broad-spectrum pyrrolidinone cephalosporin, against gram-negative nonfermenters. *Antimicrob. Agents Chemother.* 46 871–874. 10.1128/aac.46.3.872-875.2002 11850276 PMC127485

[B226] Zhang K. Zhou X. Du P. Zhang T. Cai M. Sun P. (2017). Oxidation of beta-lactam antibiotics by peracetic acid: reaction kinetics, product and pathway evaluation. *Water Res.* 123 153–161. 10.1016/j.watres.2017.06.057 28662397

